# Long-term response to autologous anti-CD19 chimeric antigen receptor T cells in relapsed or refractory B cell acute lymphoblastic leukemia: a systematic review and meta-analysis

**DOI:** 10.1038/s41417-023-00593-3

**Published:** 2023-02-07

**Authors:** Magdi Elsallab, Moataz Ellithi, Susanne Hempel, Hisham Abdel-Azim, Mohamed Abou-el-Enein

**Affiliations:** 1 Harvard-MIT Center for Regulatory Science, Harvard Medical School, Boston, MA USA; 2 Cellular Immunotherapy Program, Cancer Center, Massachusetts General Hospital, Boston, MA USA; 3 USC/CHLA Cell Therapy Program, University of Southern California, and Children’s Hospital Los Angeles, Los Angeles, CA USA; 4 Fred and Pamela Buffet Cancer Center, University of Nebraska Medical Center, Omaha, NE USA; 5 Southern California Evidence Review Center, University of Southern California, Los Angeles, CA USA; 6 Loma Linda University School of Medicine, Cancer Center, Children Hospital and Medical Center, Loma Linda, CA USA; 7 Division of Medical Oncology, Department of Medicine, Keck School of Medicine, University of Southern California, Los Angeles, CA USA; 8 Department of Stem Cell Biology and Regenerative Medicine, Keck School of Medicine, University of Southern California, Los Angeles, CA USA

**Keywords:** Gene therapy, Drug development, Haematological cancer

## Abstract

Chimeric Antigen Receptor (CAR) T cell therapy is an effective treatment approach for patients with relapsed or refractory acute lymphoblastic leukemia (R/R B-ALL). However, identifying the factors that influence long-term response to this therapy is necessary to optimize patient selection and treatment allocation. We conducted a literature review and meta-analysis to investigate the use of autologous anti-CD19 CAR T cell therapy in both pediatric and adult patients with R/R B-ALL, using several databases including MEDLINE, Cochrane Central, ScienceDirect, Web of Science, Journals@Ovid, Embase, and clinicaltrial.gov. A total of 38 reports were analyzed, which enrolled 2134 patients. Time-to-event endpoints were estimated using reconstructed patient survival data. The study explored key modulators of response, including costimulatory domains, disease status, age, and lymphodepletion. The median overall survival and event-free survival were 36.2 months [95% CI 28.9, NR] and 13.3 months [95% CI 12.2, 17], respectively. The overall response rate was 76% [95% CI 71, 81]. The use of 4-1BB costimulatory domain in the CAR construct, administration of low-dose cyclophosphamide lymphodepletion, and pretreatment morphologic remission were associated with better overall survival, with hazard ratios of 0.72, 0.56, and 0.66, respectively. Morphologic remission and 4-1BB domain were associated with better event-free survival, with hazard ratios of 0.66 and 0.72, respectively. These findings suggest that CAR T cell therapy may offer long-term benefits to patients with R/R B-ALL. However, further research is needed to optimize patient selection and better understand the impact of various factors on the outcome of CAR T cell therapy.

## Introduction

Acute lymphoblastic leukemia (ALL) is the most prevalent type of pediatric cancer, accounting for approximately 25% of childhood cancers [1, 2]. Despite significant advancements in treatment, the relapse rate remains high (15-20% for children) [3]. Patients with relapsed or refractory (R/R) B-ALL have a much lower cure rate with an estimated 20% overall 5-year survival [3–5]. Furthermore, adults with R/R ALL historically have a poor prognosis, with cure rates below 40%, largely due to associated high-risk features [6, 7]. Chimeric Antigen Receptor (CAR) T cell therapy has been established as an effective treatment for refractory or relapsed hematological malignancies, including B-ALL [8–12]. CAR T cells are genetically engineered to express a synthetic receptor which binds to tumor antigens through a single-chain variable fragment (scFv). The scFv recognizes and binds to specific surface molecules on target tumor cells, leading to CAR-mediated cytotoxicity. Various CAR designs are being studied, with CD19 being the most commonly targeted antigen, and CD28 and 4-1BB being the most widely used costimulatory domains [13].

CD19 CAR T cell has demonstrated complete remission rates as high as 90% in R/R B-ALL patients [12]. The U.S. Food and Drug Administration (FDA) approved tisagenlecleucel for pediatrics and young adults with R/R B-ALL in 2017 [14], and more recently brexucabtagene autoleucel for adult patients aged 18 or older [15]. While these therapies have shown significant early responses in pivotal trials, their primary efficacy endpoints were based on response rates [9–11]. The FDA oncologic drugs advisory committee recommends the use of patient survival or quality of life as the primary endpoints for measuring the clinical benefits of cancer drugs and biologics [16]. Response rates, however, are not always closely related to survival or quality of life [17]. Moreover, pricing and reimbursement decisions for such therapies often hinge on the long-term outcomes of the treatments [18–21]. Thus, there is a critical need for research on the long-term efficacy of CAR T cells as a single-line treatment for R/R ALL to inform clinical and health policy decisions.

Here, we conducted a systematic review of CD19-specific CAR T cell studies in pediatric and adult patients with R/R B-ALL. We analyzed patient survival data from published Kaplan-Meyer curves to calculate overall survival and event-free survival. Additionally, we conducted a meta-analysis of the response rates and adverse events associated with the treatment. We also used multivariate Cox regression models to evaluate the influence of factors such as costimulatory domain, disease status prior to treatment, lymphodepletion regimen, study design, and patient age on treatment outcomes.

## Methods

### Search strategy

The study protocol was registered on the Open Science Framework (OSF) [22]. We conducted a literature search on MEDLINE, Cochrane Central, ScienceDirect, Web of Science, Journals@Ovid, Embase, and clinicaltrial.gov for published studies on CAR T cell therapy in patients with relapsed or refractory B-ALL until January 7th, 2022. A research librarian assisted in the development of the search strategies (Table S1). Two independent reviewers (ME and MOE) screened the citations, and potentially relevant publications were obtained and evaluated against pre-set and detailed eligibility criteria. Any disagreement were resolved by discussions among the reviewers and a third reviewer (MA) as required.

### Study eligibility

We used the PICO framework to define our research question and establish inclusion and exclusion criteria for our study (Table S2). We included clinical trials and real-world reports on the efficacy and safety of anti-CD19 CAR T cell therapy for adult and pediatric patients with relapsed or refractory B-ALL. We excluded studies on allogeneic CAR T cells, CAR T cell and hematopoietic stem cell transplant combination therapy and CAR T cell with other treatments such as PD-1 inhibitors. Studies with less than 3 patients and non-English language reports were also excluded. To avoid duplication, we used the clinical trial identifiers to consolidate multiple reports on the same trial and prioritized the one with the most recent data and longest follow-up.

### Outcomes definitions

The primary outcomes of the study were overall survival and event-free survival after CAR T cell infusion. Overall survival was defined as the time from the infusion of CAR T cells to death from any cause, and event-free survival was defined as the duration from the time of infusion to relapse or death from any cause. The median survival time and survival at 1, 2-, and 5-year intervals were also calculated. Secondary outcomes include the response rates and adverse event rates. The overall response rate was defined as the proportion of patients who had a Complete Response (CR) or CR with incomplete hematologic recovery at the first disease evaluation after anti-CD19 CAR T cell infusion. CR was defined as less than 5% blast cells in the bone marrow with the restoration of normal hematopoiesis. Minimal residual disease negativity was defined as less than 0.01% blast cells in the bone marrow by either molecular methods or flow cytometry. Safety endpoints included the incidence of any grade of cytokine release syndrome (CRS) and neurotoxicity at any time after anti-CD19 CAR T-cell infusion. Treatment-related deaths were also evaluated, which were identified as deaths that are reported by authors as being related to CAR T cell product infusion.

### Risk of bias assessment

To evaluate the quality of the evidence and the validity of the results obtained from the included reports, we performed a detailed assessment of the risk of bias (RoB). As CAR T cells are typically tested in small single-arm trials, we used a RoB tool specifically designed for case series and case reports developed by Murad et al. [23]. The tool assessed four domains: selection, ascertainment, causality, and reporting. Additionally, we also used selected questions from the Cochrane RoB tool (selection, performance, detection, attrition, reporting bias) as relevant to the study design (Table S3) [24]. The reports that met the inclusion criteria were independently reviewed by two reviewers (ME and MOE) and any discrepancies were resolved through discussion.

### Subgroups

We defined subgroups to examine potential modification of treatment effects by study-level variables such as age of participants, disease morphology, CAR construct design, cyclophosphamide dosage, and type of study. We categorized populations as pediatric/young adult or mixed population based on the upper and lower bound of the age range. Pediatric/young adult group was defined as reports that included only participants who are 25 years old or younger. Whereas the mixed-age group encompassed reports that included participants of any age. Reports were stratified based on the used costimulatory domain into 4-1BB or CD28 and according to the disease status of participants at the time of infusion. We defined morphologic disease as more than 50% of the participants having a bone marrow blast count of 5% or more prior to infusion, while morphologic remission as having more than 50% of participants in morphologic remission. The dose of cyclophosphamide for lymphodepletion was used to categorize the studies into high dose (>1,500 mg/m2 total dose) and low dose (<1,500 mg/m2 total dose). Finally, we categorized reports into clinical trials and real-world data (RWD). Reports were considered RWD if they were generated from repositories that collected data on the approved CAR T cell products (commercial use products) in a retrospective or prospective manner outside the context of a clinical trial.

### Statistical analysis

A generalized linear mixed-effects model was fitted using R studio software’s meta-package version 4.18.2 to estimate the effect sizes of CD19 CAR T cell therapy [25]. Forest plots were generated using the meta-package. Heterogeneity was expressed using I-squared statistics [26]. We conducted a sensitivity analysis using the Intention-To-Treat (ITT) population to estimate the change in response rates due to dropouts from the studies while waiting for CAR T cell infusion. Cochran’s Q test was used to test for heterogeneity between subgroups for potential effect modification by each study-level variable [26]. Publication bias was evaluated using Peters’ regression and inspection of the funnel plots to test for asymmetry [27].

To estimate time-to-event endpoints (overall survival and event-free survival), data were extracted using digitizer software (available at: https://automeris.io/WebPlotDigitizer/). Kaplan Meier curves were fed into the software and the points on the curves were manually selected to retrieve coordinates of each point. These outputs were then fed into the IPDfromKM software to reconstruct patient survival data from the curves [28]. We assessed the accuracy of the reconstruction by examining the values of root mean squared error (RMSE), mean absolute error, max absolute error, and the p-value of the Kolmogorov-Smirnov test [28]. The data were pooled from all studies to estimate median overall survival, median event-free survival, the 12-, 24- and 60-month survival probability and 95% confidence intervals (CI) using the survival package in R [29]. Kaplan Meier curves were generated using the ggplot2 package in R [30]. We used log-rank test to compare the survival distributions of different study-level variables on overall survival and event-free survival using the survival package in R.

To assess the impact of study-level variables on survival outcomes, hazard ratios were calculated using univariate Cox proportional hazard models [31]. A stepwise selection process, incorporating a significance level of 0.15 for entry and 0.05 for retention, was then used to determine which effect modifiers should be included in the final multivariate model. The assumptions of the Cox proportional hazard models were evaluated by examining Schoenfeld residuals. The multivariate model was visualized using the survminer package [32]. All analyses were conducted using R Studio version 1.4.1717 (RStudio: Integrated Development for R. RStudio, PBC, Boston, MA, USA).

## Results

A total of 11273 reports were retrieved, of which 298 were obtained as full-text and 54 were eligible for analysis. Sixteen reports were excluded due to high risk of bias (Table S4). The remaining 38 reports were included in the quantitative synthesis, with a total of 2134 patients, out of which 1908 received CD-19 CAR T cell products [33–51, 12, 11, 52–59, 10, 60–67] (Fig. S1). The study found no indication of potential publication bias in the primary outcomes through visual inspection of funnel plots and using Peters’ test (*p* = 0.474). The characteristics of the included reports are reported in Table 1. Only 4 of the 38 reports reported information on the race/ethnicity of the participants [10, 52, 59], with African Americans and Asians representing 6.2% and 4.3% respectively [52, 59, 60], and only two reported the percentage of Hispanic participants [52, 59]. One report listed minority groups as aggregate data which did not allow further analysis. More information on the reports can be found in tables S5, S6, and S7.

**Table 1:** Reports and patients characteristics.

**Report characteristics**	
*Report type, n (%)*	
Phase I	16 (42)
Phase I/II	12 (31)
Phase II	6 (16)
Retrospective	1 (3)
Real world	3 (8)
*Report location, n (%)*	
Asia	15 (39)
Europe	4 (11)
North America	14 (37)
Other	5 (13)
*Number of centers, median (range)*	1 (1–73)
*Survival curves, n (%)*	
Yes	25 (67)
No	13 (33)
*CRS Scale used, n (%)*	
ASTCT	5 (13)
Lee	18 (47)
MSKCC	2 (5)
Penn	7 (19)
Unreported	6 (16)
***CAR design***	
*Vector type, n %*	
Lentivirus	26 (68)
Retrovirus	7 (18)
Other	1 (3)
Unreported	4 (11)
*Costimulatory domain, n %*	
4-1BB	27 (71)
CD28	5 (13)
CD28/4-1BB	4 (11)
Unreported	2 (5)
*CAR Hinge, n %*	
CD28	5 (13)
CD8	18 (47)
IgG4	2 (5)
Unreported	13 (34)
*CAR scFV Clone, n %*	
A3B1	1 (3)
CAT13.1E10	2 (5)
FMC63	21 (55)
HI19α	1 (3)
Unreported	13 (34)
***Patient characteristics***	
Patients Enrolled, *n*	2134
Patients Infused, *n*	1908
Age, range	0.4–76
Median number of therapies, range	2–9
Previous HST, % (95% CI)	29 (23–37)
Median blast percentage, range	0.25–74

*ASTCT* The American Society for Transplantation and Cellular Therapy, *CRS* Cytokine Release syndrome, *CAR* Chimeric Antigen Receptor, *HST* Hematopoietic stem cell transplantation, *MSKCC* Memorial Sloan Kettering Cancer Center, *scFv*: single chain variable fraction.

### Effect estimates

**Fig. 1:**
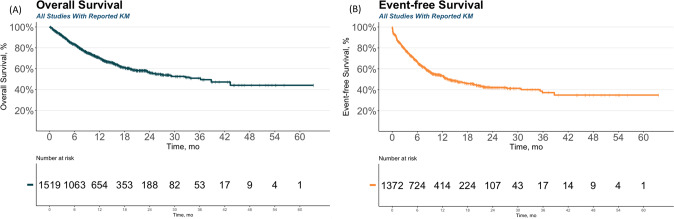
Overall survival and event-free survival of R/R B-ALL patients treated with anti-CD19 CAR T therapy. Pooled data from published Kaplan Meier curves were used to estimate (**A**) the Overall Survival (OS) of the infused population and (**B**) the Event-free survival (EFS) of the infused population.

**Fig. 2:**
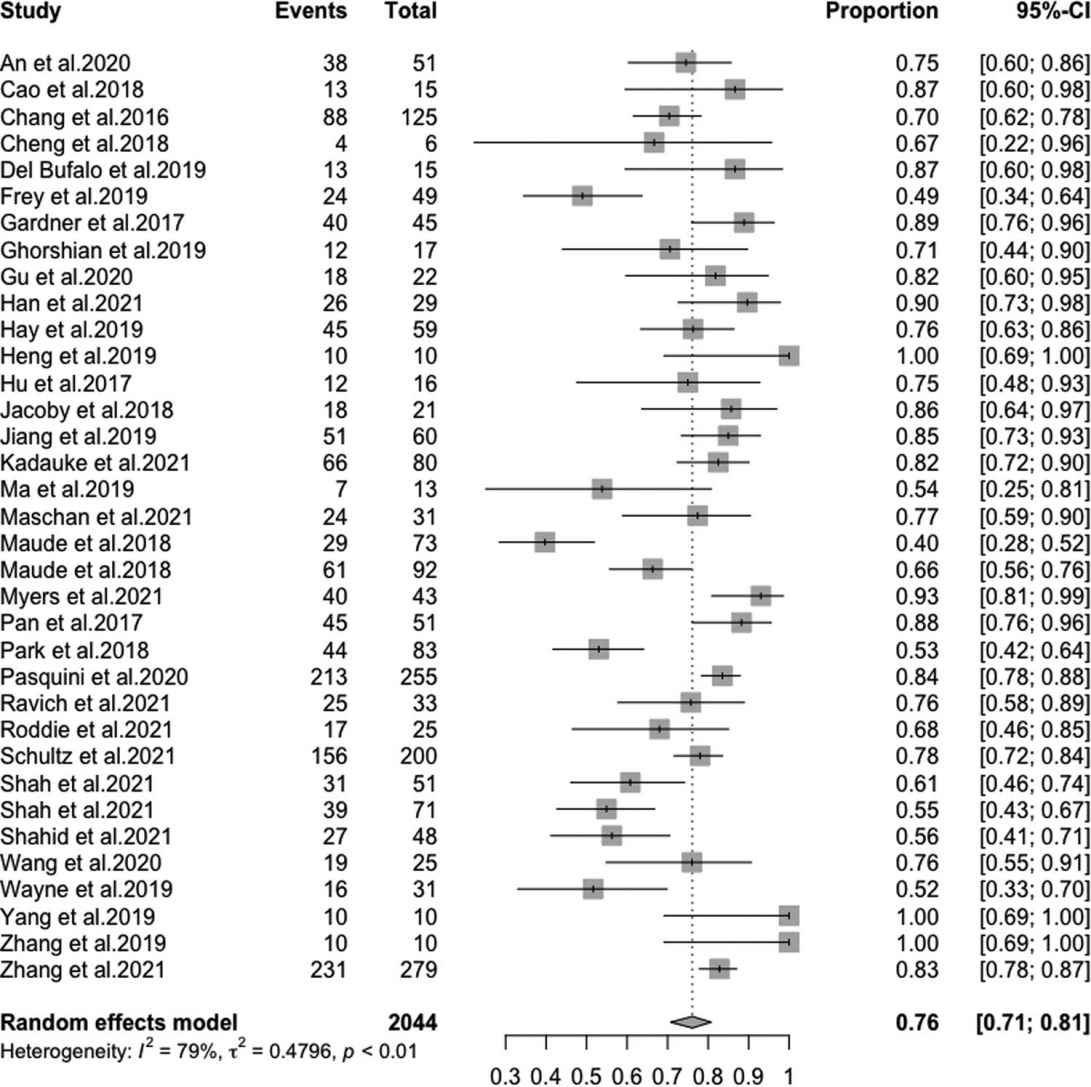
Overall response in R/R ALL patients. Forest plot of the overall response in all studies using the ITT (enrolled) population. The response rates were aggregated using a generalized linear mixed-effect model.

The study showed that the median overall survival was 36.2 months [95% CI 28.9, NR], and the median event-free survival was 13.3 months [95% CI 12.2, 17] (Fig. 1). The 12-month and 24-month overall survival rates were 70% [95% CI 67.7, 72.8] and 56.5% [95% CI 53.2, 60], while the 12-month and 24-month event-free survival rates were 53.2% [95% CI 50.3, 56.2] and 42.1% [95% CI 38.7, 45.8]. At 5 years, the overall survival and event-free survival were 44.1% [95% CI 36.3, 53.5] and 35% [95% CI 28.8, 42.5], respectively (Fig. 1). The overall response rate was 76% [95% CI 71, 81] in the ITT population (Fig. 2), and 85% in the mITT population [95% CI 82, 88] (Fig. S3). Of the responding patients, 98% [95% CI 94, 99] achieved MRD-negative remission (Fig. S4), and 26% [95% CI 20, 34] of infused patients went on to have a HSCT (Fig. S5).

Cytokine release syndrome (CRS) of any degree was reported in 83% [95% CI 76, 89] of the infused patients, while 21% [95% CI 16, 26] developed grade 3 or higher CRS (Figs. S6, S7). Neurotoxicity of any grade was reported in 30% [95% CI 24, 38] of the infused patients (Fig. S8). Of the infused patients 4% [95% CI 3, 6] suffered from treatment-related deaths. (Fig. S9).

### Treatment effect modifiers

**Fig. 3:**
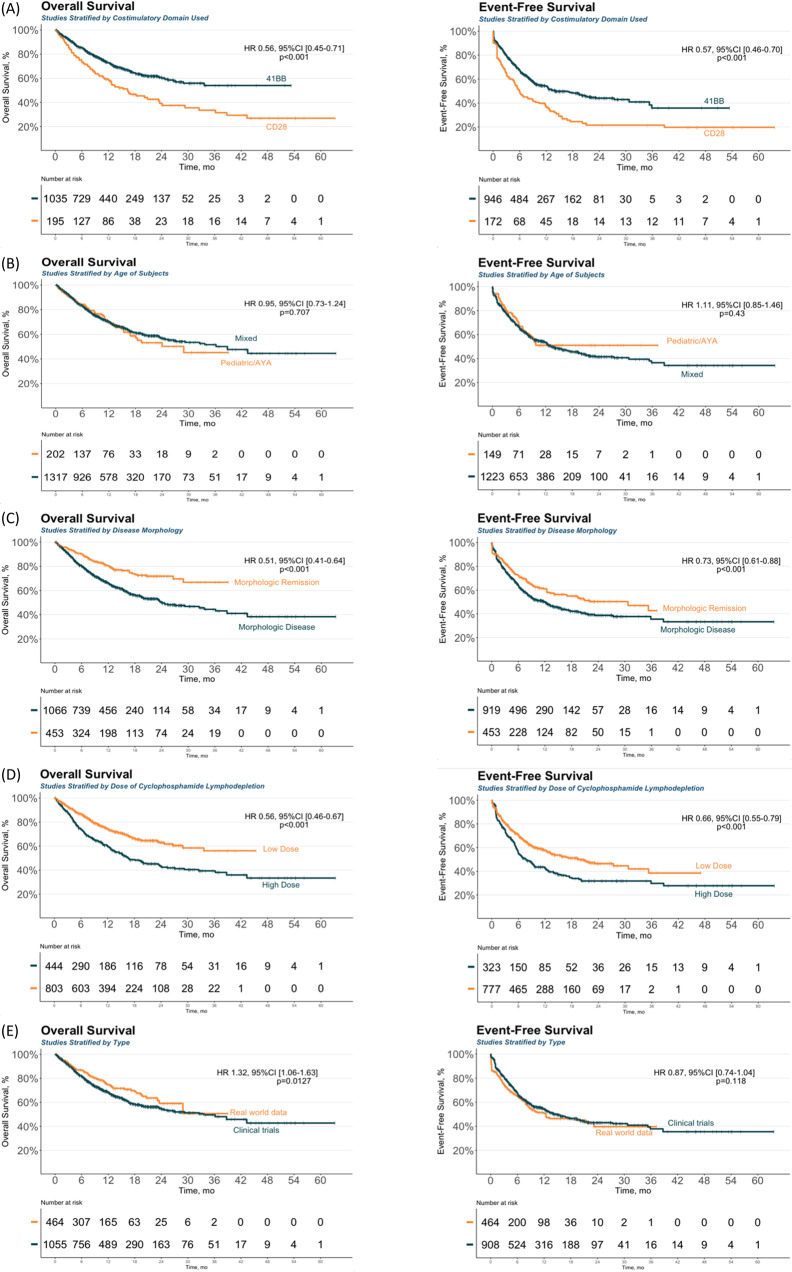
Subgroup analyses of the Overall survival and Event free survival Study level characteristics. The survival data from the pooled studies were used to estimate (**A**) the OS (right) and EFS (left) based on the co-stimulatory domain used in the study, (**B**) the OS (right) and EFS (left) based on the age range of the study population, (**C**) the OS (right) and EFS (left) based on the pretreatment disease status, (**D**) the OS (right) and EFS (left) stratified based on the dose of cyclophosphamide lymphodepletion, and (**E**) the OS (right) and EFS (left) based on the study type.

The 38 reports included in the quantitative synthesis were analyzed to determine the effect of various study-level variables on patient survival (Table 2). The univariate Cox proportional hazard analysis showed that the use of the 4-1BB signaling domain, low-dose cyclophosphamide, and being in morphologic remission at the time of infusion, were associated with better overall and event-free survival (Fig. 3, Table S8, Fig. S10). The univariate analysis of the relationship between the start date of the studies and survival outcomes found that more recent studies had better overall survival, with a hazard ratio (HR) of 0.90 [95% CI 0.85–0.94, *p* < 0.001], and better event-free survival, with a HR of 0.93 [95% CI 0.90–0.97, *p* < 0.001] (Table S8). Additionally, and real-world data reports were also found to have better overall, and event-free survival compared to clinical trials (Fig. 3E).

**Table 2:** Distribution of the studies and patients across the different subgroups and analyses.

Subgroup	Response rate analysis reports				Survival analysis reports			
	N (%)	Pt enrolled	N (%)	Pt infused	OS		EFS	
					N (%)	Pt	N (%)	Pt
Domain	29		32		23		21	
4-1BB	24 (83)	1309	27 (84)	1289	18 (78)	1035	17 (81)	946
CD28	5 (17)	257	5 (16)	202	5 (22)	195	4 (19)	172
Age group	36		38		25		22	
Pediatric/young adult (≤ 25)	11 (31)	420	11 (30)	354	5 (20)	202	4 (18)	149
Mixed (≤ 25 and/or >25)	25 (69)	1646	27 (71)	1554	20 (80)	1317	18 (82)	1223
Disease morphology	33		35		25		22	
Remission (BMB < 5%)	9 (27)	464	10 (29)	485	7 (28)	453	7 (32)	453
Disease (BMB ≥ 5%)	24 (73)	1478	25 (71)	1303	18 (72)	1066	15 (68)	919
LD dose (Cy total dose^a^)	28		30		23		20	
High (≥1500 mg/m^2^)	11 (39)	602	11 (37)	494	10 (43)	444	8 (40)	323
Low (<1500 mg/m^2^)	17 (61)	945	19 (63)	926	13 (57)	803	12 (60)	777
Report type	35		38		25		22	
Clinical trial	32 (91)	1556	35(92)	1437	22 (88)	1055	19 (86)	908
RWD	3 (9)	488	3 (8)	471	3 (12)	464	3 (14)	464

*N* number of studies, *Pt* Patients, *OS* overall survival, *EFS* Event-free survival, *BMB* bone marrow blast percentage, *LD* lymphodepletion, *Cy* cyclophosphamide, *RWD* Real world data.

^a^Dose calculated as mg/m^2^ or equivalent.

**Fig. 4:**
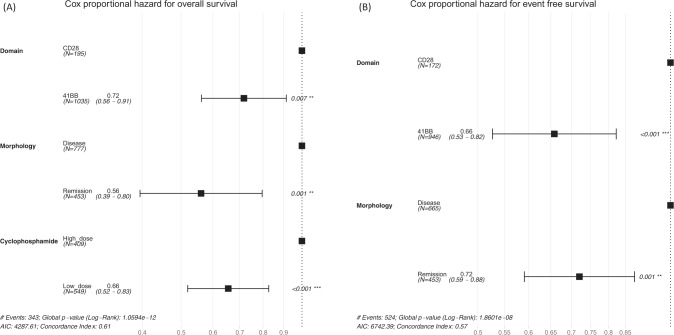
Multivariate analysis of survival. Selected study-level variables were included in a multivariate Cox regression analysis of the (**A**) overall survival and (**B**) Event-Free Survival.

The multivariate analysis of study-level variables revealed that the use of 4-1BB as a costimulatory domain in the CAR T-cell construct, administering low-dose cyclophosphamide for lymphodepletion, and patients being in morphologic remission at the time of infusion were associated with better overall and event-free survival. Specifically, the HR for death was 0.72 (*p* = 0.007) for the 4-1BB domain, 0.56 (*p* < 0.001) for low-dose lymphodepletion and 0.66 (*p* < 0.001) for morphologic remission (Fig. 4A). Similarly, the HR for relapse or death was 0.66 (*p* < 0.001) for morphologic remission and 0.72 (*p* = 0.001) for the 4-1BB domain (Fig. 4B).

The subgroup analysis of response rates found that CAR T cells using the 4-1BB domain had a higher overall response rate (78% [95% CI 72–83]) and MRD negative remission rate (99% [95% CI 96–100]) compared to those using the CD28 domain (58% [95% CI 51–63] *p* < 0.001 and 90% [95% CI 68–97] *p* = 0.018, respectively), (Table 3). The proportion of patients proceeding to HSCT was higher in the CD28 group (38.4% [95% CI 24.8–54.1]) compared to the 4-1BB group (20.5% [95% CI 15–26.7]), *p* = 0.017). No differences were detected in the rate of CRS between the two domains, but neurotoxicity rate was higher in the CD28 subgroup (*p* = 0.038).

**Table 3:** Effect modifiers of CD19 CAR T cells safety and efficacy.

Subgroup	Overall response (ITT) % (95% CI)		Overall response (mITT) % (95% CI)		Minimal residual disease negative % (95% CI)		Cytokine release syndrome (CRS) % (95% CI)		Grade 3 or higher CRS % (95% CI)		Neurotoxicity % (95% CI)	
*Domain*												
41BB	*N* = 24	78 [72; 83]	*N* = 27	86 [82; 89]	*N* = 26	99 [96; 100]	*N* = 23	86 [76; 92]	*N* = 25	23 [17; 30]	*N* = 22	29 [22; 37]
CD28	*N* = 5	58 [51; 63]	*N* = 5	74 [64; 82]	*N* = 5	90 [68; 97]	*N* = 5	83 [75; 89]	*N* = 5	23 [18; 30]	*N* = 5	46 [32; 62]
*p*-value		< 0.001*		0.0059*		0.0179		0.5834		0.9713		0.0380*
*Age group*												
Pediatric/young adult	*N* = 10	68 [56; 77]	*N* = 11	82 [72; 89]	*N* = 11	99 [93; 100]	*N* = 9	79 [66; 88]	*N* = 10	22 [14; 33]	*N* = 8	42 [33; 53]
Mixed	*N* = 25	79 [73; 84]	*N* = 27	86 [83; 89]	*N* = 25	96 [91; 98]	*N* = 24	85 [76; 91]	*N* = 26	20 [15; 27]	*N* = 22	26 [18; 35]
*p*-value		0.048*		0.3608		0.2537		0.4555		0.7932		0.0125*
*Disease morphology*												
Remission	*N* = 9	84 [80; 87]	*N* = 10	90 [83; 94]	*N* = 23	97 [91; 99]	*N* = 9	82 [61; 93]	*N* = 10	16 [13; 20]	*N* = 10	24 [16; 36]
Disease	*N* = 24	72 [65; 77]	*N* = 25	83 [78; 86]	*N* = 10	99 [92; 100]	*N* = 23	84 [76; 90]	*N* = 25	23 [17; 30]	*N* = 18	37 [28; 46]
*p*-value		< 0.001*		0.0588		0.2831		0.8248		0.0720		0.0948
*LD dose*												
High	*N* = 11	66 [56; 75]	*N* = 11	79 [71; 86]	*N* = 11	96 [89; 99]	*N* = 9	80 [69; 88]	*N* = 11	25 [14; 39]	*N* = 9	32 [24; 41]
Low	*N* = 17	81 [74; 86]	*N* = 19	87 [83; 91]	*N* = 18	98 [94; 100]	*N* = 17	86 [73; 93]	*N* = 18	19 [15; 24]	*N* = 17	32 [21; 45]
*p*-value		0.0093*		0.0413*		0.3778		0.4142		0.3843		0.9831
*Report type*												
Clinical trial	*N* = 32	76 [70; 81]	*N* = 35	86 [82; 89]	*N* = 33	97 [93; 99]	*N* = 30	85 [78; 90]	*N* = 33	21 [16; 27]	*N* = 27	32 [24; 40]
RWD	*N* = 3	80 [77; 84]	*N* = 3	83 [80; 87]	*N* = 3	100 [98; 100]	*N* = 3	59 [53; 64]	*N* = 3	18 [15; 22]	*N* = 3	21 [15; 31]
*p*-value		0.1242		0.5456		0.0377*		0.0001*		0.4075		0.1061

*LD* lymphodepletion, *RWD* real world data.

* Statistically significant.

The subgroup analysis also showed that reports including pediatric/young adult patients had a higher incidence of neurotoxicity (*p* = 0.0097). Reports that had a higher proportion of patients in morphologic remission before CAR T cell infusion had a better overall response rate (*p* < 0.001), with no significant difference in the incidence of CRS or neurotoxicity (*p* = 0.414 and 0.983, respectively). Reports that used lower doses of cyclophosphamide also had a better overall response rate. Furthermore, RWD reports had a lower incidence of CRS compared to clinical trials (*p* < 0.001) (Table 3).

## Discussion

In this meta-analysis, we investigated the long-term outcomes and safety of CAR T cell therapy in r/r ALL using data from 2134 patients and outlined factors that may affect the response to this type of treatment. Our analysis indicates that while most patients elicit an initial response to CAR T cells, the 5-year survival suggest that more than half of these patients might experience relapse after treatment. Both patient and product characteristics appear to influence the long-term outcomes of CAR T cell therapy. We observed worse survival in trials with high number of patients with morphologic disease before treatment, which is consistent with other studies [55, 68]. Recent analyses suggest that pre-existing CD19^neg^ clones may contribute to relapse after CAR T cell therapy [69]. This is noteworthy, as about half of relapses in ALL patients treated with CAR T cells are CD19^neg^ [11, 39]. Additionally, the cellular composition and pharmacokinetics of the CAR T cell product and the expansion of certain subpopulations may also affect the response and contribute to patient relapse [70, 71].

The costimulatory domain used in the CAR T cell product can also have a significant impact on the long-term outcome of treatment. Published studies suggest that the CD28 costimulatory domain induces differentiation to an effector-like cell phenotype with higher production of cytokines compared to 4-1BB, which induces differentiation to a memory-like phenotype of the CAR T cells [72, 73]. Our findings provide further clinical confirmation of these prior studies and indicate that CAR T cells with 4-1BB costimulatory domains have a more sustained response compared to those with CD28 domains, which may be related to the differences in the differentiation and persistence of T cells [70, 72, 73]. This significant difference persisted even after accounting for variations in patient age and could explain the higher proportion of patients in the CD28 group proceeding to transplantation. Another product characteristic that can influence the effectiveness of CAR T cell therapy is the quality of the starting material used to manufacture the product. Studies have suggested that patient age may impact the quality of the starting material, with adults potentially having worse outcomes compared to children/young adults [74, 75]. In contrast, our subgroup analysis did not show that including adult patients in the study population had a negative impact on long-term outcomes after CAR T cell therapy.

We also observed that the use of low-dose cyclophosphamide lymphodepletion before CAR T cell infusion was associated with better overall survival. Currently, evidence regarding the optimal dose of lymphodepletion remains largely inconclusive. While some clinical studies suggested that high-dose cyclophosphamide improves responses to CAR T cells, other studies suggest that aggressive lymphodepletion and bridging therapy may not offer additional clinical benefits and increase toxicities [10, 11, 76–78]. Furthermore, aggressive chemotherapy before CAR T cell infusion might affect the activation and expansion of CAR T cells by modulating the target density [79–81]. A more granular analysis that accounts for multiple confounding factors is needed to fully understand the impact of patient and product characteristics on long-term survival.

We observed that the majority of patients experienced CRS of any grade and a third experienced neurotoxicity of any grade. While previous studies have suggested a link between disease burden and the severity of these adverse events [82, 83], this analysis did not find a significant difference based on disease burden. Despite the high incidence of serious adverse effects, the reported rate of treatment-related mortality was relatively low [84]. Furthermore, RWD reports showed lower rates of CRS, which could be attributed to early recognition and better management. Overall, there is a continued need for optimization of next-generation CAR T cell designs to improve safety profiles and minimize toxicities.

The accurate and detailed reporting of patient characteristics is crucial to assess the generalizability of study results [85]. Our analysis highlighted a few areas where the reporting of clinical data could be improved, such as providing time-to-event data and information on the number of patients at risk and censoring in survival curves. Additionally, we found that the racial and ethnic backgrounds of recruited patients were often not reported or underrepresented. This aligns with previous research, which suggests that subjects of color and minority descent are frequently underrepresented in CAR T cell trials [86]. It is important to ensure diversity in clinical trials to consider the potential differences in responses among different groups [87, 88].

Limitations in our report are largely inherit to meta-analysis. The lack of comparator arms limits our ability to compare the effectiveness of CAR T cell therapies to other treatment options. Additionally, the lack of patient-level data restricts our ability to analyze a wider range of effect modifiers or to use matching methods to compare patients across different studies. Furthermore, the use of different scoring systems to report safety outcomes may make it difficult to compare these outcomes across studies. New methods for meta-analysis of single-arm trials, such as network meta-analysis and matched-adjusted indirect comparison, are being developed to overcome some of these limitations. However, these methods would also require individual patient data to be used effectively.

## Conclusion

Long-term outcomes of CAR T cell therapy, despite being an important measure of treatment efficacy, remain insufficiently reported. Our analysis indicates that CAR T cells can offer long-term benefits to patients with R/R B-ALL, who otherwise have a low overall 5-year survival rate. However, the costimulatory domain used in the CAR T cells, the disease status of the patient at the time of infusion, and cyclophosphamide dose of lymphodepletion had a major impact on patient outcomes and the risk of relapse. Further research into these effect modifiers, using well-controlled studies and improved reporting, could help to optimize patient selection and improve the overall effectiveness of CAR T cell therapy. These efforts are expected to ensure administering CAR T cells that can increase patient survival, has lower incidence of toxicity, and ultimately lower the cost of patient treatment.

## Supplementary information

Supplemental Material

## Data Availability

The datasets generated during and/or analysed during the current study are available from the corresponding authors on request.
