# Supplementary material for: Long-term response to autologous anti-CD19 chimeric antigen receptor T cells in relapsed or refractory B cell acute lymphoblastic leukemia: a systematic review and meta-analysis
Source: Cancer Gene Ther. 2023 Feb 7;30(6):845–54. doi: 10.1038/s41417-023-00593-3 (PMC10281866; doi:10.1038/s41417-023-00593-3)
Supplement: Supplementary file 1 — Supplemental Material [file 41417_2023_593_MOESM1_ESM.pdf]

Table S1. Search terms

Domain	("Leukemia"[MeSH] OR "Acute lymphoblastic leukemia" OR "Acute lymphoblastic leukaemia" OR "Acute lymphoblastic leukemias" OR "Acute lymphoblastic leukaemias" OR "Leukemia, Acute Lymphoblastic" OR "Leukaemia, Acute Lymphoblastic" OR "Leukemias, Acute Lymphoblastic" OR "Leukaemias, Acute Lymphoblastic")
Determinant	(CAR[Title/Abstract] OR CART[Title/Abstract] OR CART19[Title/Abstract] OR CAR[Text Word] OR CART[Text Word] OR CART19[Text Word] OR "Receptors, Chimeric Antigen"[Mesh] OR "Immunotherapy, Adoptive"[Mesh] OR (adoptive AND (immunotherapy OR immunotherapies)) OR (chimeric AND antigen AND (receptor OR receptors)) OR "CD19-specific chimeric antigen receptor" [Supplementary Concept])

Table S2. PICO criteria

Population	Pediatric and Adult with Relapsed or Refractory Acute lymphocytic leukemia
Intervention	Autologous CD19 Chimeric Antigen receptor T cells
Comparison	NA
Outcome	• Overall Survival<br>• Event Free survival<br>• Overall response<br>• Cytokine release syndrome<br>• Neurotoxicity

Table S3. Risk of Bias Assessment Tool

Selection	**1.1. Was the target population clearly defined with inclusion and exclusion criteria? (Y/PY/N/PN/NI)**<br>**1.2. Was enrollment defined by a period of time? (Y/PY/N/PN/NI)**<br>**1.3. Was any of the enrolled patients excluded pre-treatment? (Y/PY/N/PN/NI)**<br>**1.4. If yes, was the reason of the exclusion indicated? (Y/PY/N/PN/NI)**<br>**1.5. Is it possible that the exclusion of the cases was based on their unfavorable pre-treatment condition? (Y/PY/N/PN/NI)**
Ascertainment	**2.1. Was the treatment plan predefined? (Y/PY/N/PN/NI)**<br>**2.2. Was the dose of the treatment predefined? (Y/PY/N/PN/NI)**<br>**2.3. Were co-treatments indicated? (Y/PY/N/PN/NI)**<br>**2.4. Was the method of measuring the outcome predetermined? (Y/PY/N/PN/NI)**<br>**2.5. Was outcome evaluated in all enrolled patients? (Y/PY/N/PN/NI)**<br>**2.6. Was the outcome evaluated by a standardized score or method? (Y/PY/N/PN/NI)**
Causality	**3.1. Were other treatments that may explain the observation outcome ruled out? (Y/PY/N/PN/NI)**<br>**3.2. Was follow-up long enough for outcomes to occur? (Y/PY/N/PN/NI)**<br>**3.3. Was there a dose response relationship? (Y/PY/N/PN/NI)**
Reporting	**4.1. Was the data for all the outcomes reported? (Y/PY/N/PN/NI)**<br>**4.2. Were data for this outcome available for all, or nearly all, participants? (Y/PY/N/PN/NI)**<br>**4.3. Is there evidence that the result was not biased by missing data? (Y/PY/N/PN/NI)**<br>**4.4. Is it likely that missingness in the outcome depended on its true value? (Y/PY/N/PN/NI)**

Table S4. Risk of Bias Assessment for the selected studies

Study	*Selection*	*Ascertainment*	*Causality*	*Reporting*	*Final Score*
**An_Nature_2020**	Low	Low	Low	Low	Low
**Cao_AJH_2018**	Low	Low	Low	Low	Low
**Cao_Blood_2019**	Some Concern	Low	High	Some Concern	**High**
**Chang_Blood_2016**	Some Concern	Low	Low	Low	Low
**Cheng_Mol_ther_2020**	Low	Low	High	Low	Some Concern
**Chong_Blood_2018**	High	High	High	High	**High**
**Del_Bufalo_Blood_2019**	Low	Low	Low	Low	Low
**Deng_Blood_2019**	Low	Low	High	High	**High**
**Dourthe_Blood_2019**	High	Low	Some Concern	Some Concern	**High**
**Frey_JCO_2018**	High	Low	High	High	**High**
**Frey_JCO_2020**	Low	Low	Low	Low	Low
**Gardner_Blood_2017**	Low	Low	Low	Low	Low
**Gauthier_Blood_2018**	Low	Some Concern	High	High	**High**
**Ghorashian_Nat_2019**	Low	Low	Low	Low	Low
**Gu_J_hem_onc_2020**	Low	Low	Low	Low	Low
**Han_CanBio_2021**	Low	Low	High	Low	Low
**Hay_Blood_2019**	Low	Low	Low	Low	Low
**Heng_CCR_2020**	Low	Low	High	Low	Low
**Hu_CCR_2017**	Low	Low	Low	Low	Low
**Jacoby_AJH_2018**	Low	Low	Low	Low	Low
**Jiang_AJH_2019**	Low	Low	Low	Low	Low
**Kadauke_JCO_2021**	Low	Low	Low	Low	Low
**Khaled_Blood_2018**	High	Low	High	High	**High**
**Lee_EHA_2017**	High	Low	High	High	**High**
**Ma_Hem_onc_2019**	Low	Low	Low	High	Some Concern
**Maschan_nat_2021**	Low	Low	Low	Some Concern	Low
**Maude_Blood_2017**	Low	Low	Low	Low	Low
**Maude_EHA_2018**	Low	Low	Low	Low	Low
**Maude_NEJM_2014**	Low	Low	Low	Low	Low
**Maude_NEJM_2018**	Low	Low	Low	Low	Low
**Myers_JCO_2021**	Low	Low	Low	Low	Low
**Ortíz_Mol_ther_2020**	Low	Low	Low	Low	Low
**Pan_EHA_2016**	High	Low	High	High	**High**
**Pan_Leukemia_2017**	Low	Low	Low	High	Some Concern
**Park_NEJM_2018**	Low	Low	Low	Low	Low
**Pasquini_Blood_adv_2020**	Low	Low	Low	Low	Low
**Ravich_transpl_2021**	Low	Low	Low	Low	Low
**Roddie_JCO_2021**	Low	Low	Low	Low	Low
**Sanfang_Eur_J_Imm_2019**	High	High	Low	High	**High**
**Schultz_JCO_2021**	Low	Low	Low	Low	Low
**Shah_JCO_2021**	Low	Low	Low	Low	Low

**Shah_lancet_2021**	Low	Low	Low	Low	Low
**Shahid_transplnt_2021**	Low	Low	Low	Low	Low
**Shen_Ped_hem _2020**	High	High	Low	High	**High**
**Talleur_CLML_2019**	High	Low	High	High	**High**
**Tang_Blood_2018**	Low	High	High	High	**High**
**Trede_clinicaltrial.gov**	Low	High	High	High	**High**
**Wang_BJH_2020**	Low	Low	Low	Low	Low
**Wayne_EHA_2019**	Low	Low	Low	Low	Low
**Weng_J_hem_onc_2018**	Low	Some Concern	High	Some Concern	**High**
**Xiao_JCO_2017**	Low	Low	High	High	**High**
**Yang_Blood_2019**	Low	Low	High	Low	Low
**Zhang_Blood_2019**	Low	Low	Low	Low	Low
**Zhang_CII_2021**	Low	Low	Low	Low	Low

Flow Diagram of the Study Selection Process

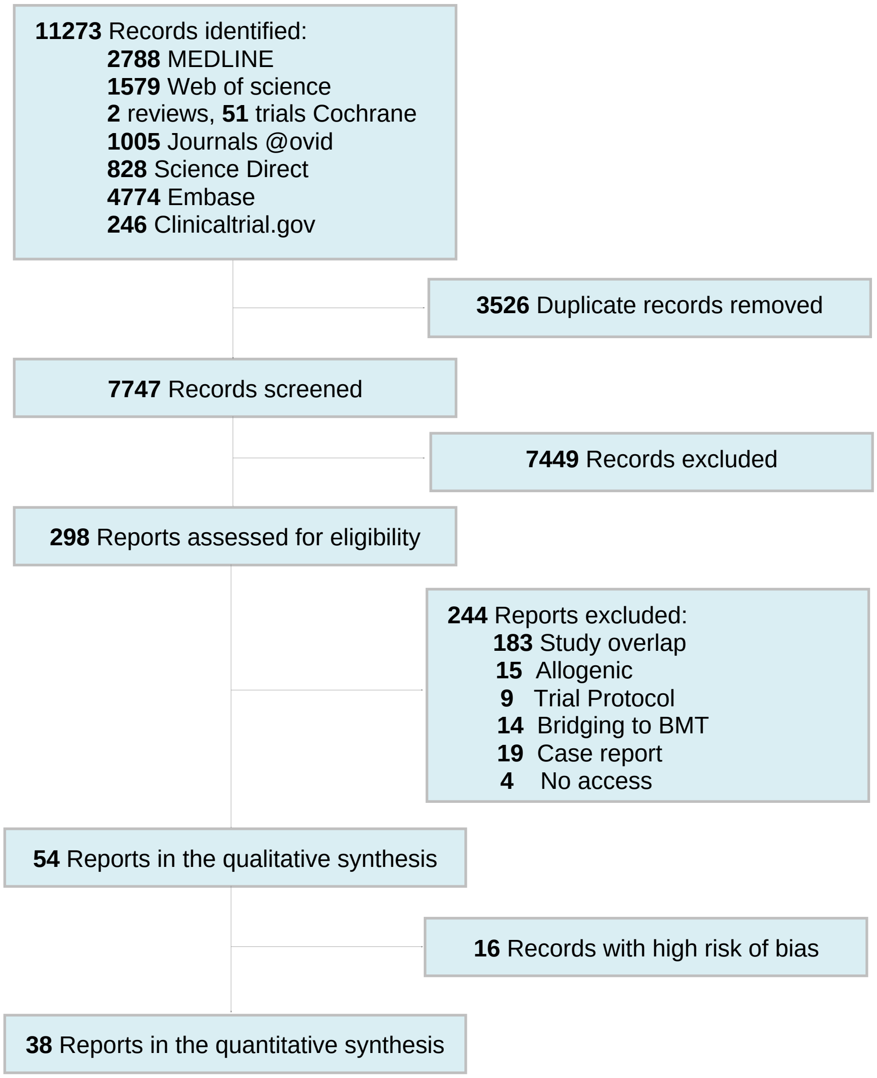

Figure S1. Study Selection Flowchart

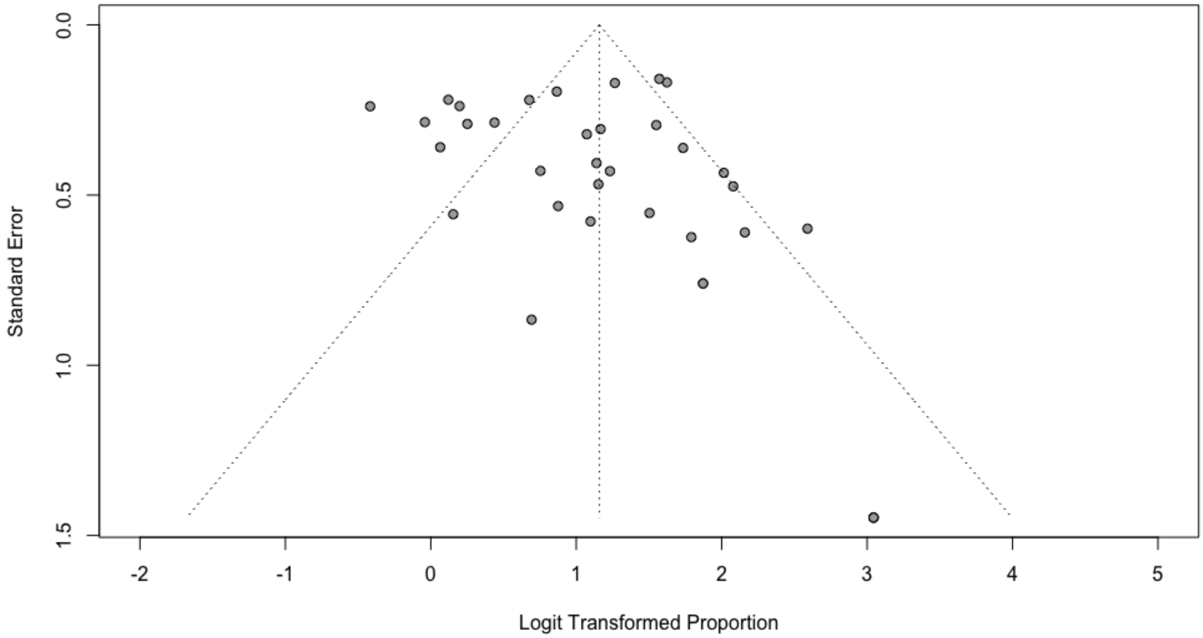

Figure S2. Funnel plot of the overall response in the enrolled population

Table S5 *Evidence Table of the included studies*

	**Intervention**					**Participants**									
	Registration number	Vector	Hinge	Domain	N enrolled	Age, median (Range)	Previous transplantation (%)	Median blast percentage	Detectable disease (%)	Median number of prior therapies	Minimal residual disease-negative (%)	Central Nervous System disease (%)	Extramedullary disease (%)	Unfavorable Cytogenetics (%)	Philadelphia Chromosome Positive (%)
An et al (n=47)(1)	NCT02735291	Retrovirus	IgG4	4-1BB	51	22 (3-72)	9 (19%)	NA	NA	NA	NA	6 (13%)	13 (28%)	29 (62%)	11 (23%)
Cao et al (n=15)(2)	NCT02782351	Lentivirus	CD8	4-1BB	15	14 (3-57)	NA	60.5	14 (93%)	9	1 (7%)	4 (27%)	5 (33%)	NA	NA
Chang et al (n=102)(3)	NA	Lentivirus	NA	CD28/4-1BB	125	NA (2-70)	NA	14.5	NA	NA	NA	NA	NA	NA	NA
Cheng et al (n=6)(4)	NCT02685670	Retrovirus	CD8	CD28/4-1BB	6	26 (7-45)	NA	0.25	2 (33%)	2	NA	3 (50%)	4 (67%)	NA	NA
Del Bufalo et al (n=15)(5)	NA	Retrovirus	NA	4-1BB	15	10 (4-24)	9 (60%)	9.7	9 (60%)	NA	NA	NA	NA	7 (47%)	1 (7%)
Frey et al (n=35)(6)	NCT02030847; NCT01029366	Lentivirus	CD8	4-1BB	49	34(21 - 70)	13 (37%)	NA	27 (77%)	3	NA	NA	NA	NA	3 (9%)
Gardner et al (n=43)(7)	NCT02028455	Lentivirus	NA	4-1BB	45	12 (1-25)	28 (65%)	NA	27 (62%)	NA	NA	9 (21%)	NA	NA	8 (19%)
Ghorashian et al (n=14)(8)	NCT02443831	Lentivirus	CD8	4-1BB	17	9 (1-19)	10 (71%)	1	4 (23.5%)	4	10 (71%)	2 (14%)	NA	12 (86%)	1 (7%)
Gu et al (n=20)(9)	NCT02975687	Lentivirus	NA	4-1BB	22	18 (3–52)	NA	35.6	18 (82%)	2	NA	NA	2 (10%)	20 (100%)	20 (100%)
Han et al (n=29)(10)	NCT02924753; NCT03101709	Lentivirus	CD8	4-1BB	29	22 (2-60)	NA	32.5	25 (86%)	NA	0 (0%)	NA	NA	NA	NA
Hay et al (n=53)(11)	NCT01865617	Lentivirus	IgG4	4-1BB	59	39 (20-76)	23 (43%)	28	34 (58%)	3	14 (26%)	5 (9%)	18 (34%)	41 (77%)	11 (21%)
Heng et al (n=10)(12)	NCT02349698	Lentivirus	CD8	4-1BB	10	16 (5-40)	2 (20%)	4.5	5 (50%)	6.5	0 (0%)	3 (30%)	NA	9 (90%)	1 (10%)
Hu et al (n=14)(13)	ChiCTR-OCC-15007008	Lentivirus	NA	4-1BB	16	32 (7-57)	5 (36%)	64	13 (93%)	NA	NA	4 (29%)	6 (43%)	6 (43%)	4 (29%)
Jacoby et al (n=20)(14)	NCT02772198	Retrovirus	NA	CD28	21	11 (5-48)	10 (50%)	50	12 (60%)	4	NA	6 (30%)	8 (40%)	NA	1 (5%)
Jiang et al (n=58)(15)	NCT02965092	Lentivirus	CD8	4-1BB	60	28 (10-65)	3 (5%)	12.1	37 (64%)	2	6 (10%)	NA	NA	18 (31%)	7 (12%)
Kadauke et al (n=70)(16)	NCT02906371	Lentivirus	CD8	4-1BB	80	11 (1-29)	25 (36%)	NA	26 (37%)	NA	27 (39%)	9 (13%)	NA	26 (37%)	NA
Ma et al (n=10)(17)	NCT02963038	NA	CD28	4-1BB	13	7 (3-13)	0 (0%)	59.2	9 (90%)	4	NA	NA	NA	NA	0 (0%)
Maschan et al (n=27)(18)	NCT03467256	Lentivirus	CD8	4-1BB	31	10 (1-20)	13 (48%)	13	18 (67%)	3	0 (0%)	NA	NA	NA	NA
Maude et al (n=22)(19)	NA	Lentivirus	NA	4-1BB	NA	NA (2-24)	NA	NA	NA	NA	NA	7 (32%)	NA	NA	NA
Maude et al (n=58)(20)	NCT02228096 (ENSIGN)	Lentivirus	CD8	4-1BB	73	12 (3-25)	26 (45%)	69	58 (100%)	3	NA	7 (12%)	NA	NA	NA

Maude et al (n=30)(21)	NCT01626495; NCT01029366	Lentivirus	CD8	4-1BB	NA	14 (5-60)	18 (60%)	NA	24 (80%)	NA	5 (17%)	2 (7%)	NA	8 (27%)	NA
Maude et al (n=75)(22)	NCT02435849 (ELIANA)	Lentivirus	CD8	4-1BB	92	11 (3-23)	46 (61%)	74	75(100%)	3	NA	11 (15%)	NA	28 (37%)	NA
Myers et al (n=41)(23)	NCT02374333	Lentivirus	NA	4-1BB	43	10 (2-29)	16 (39%)	NA	9 (22%)	NA	25 (61%)	1 (2%)	NA	16 (39%)	NA
Ortíz et al (n=38) (24)	NCT03144583	Lentivirus	CD8	4-1BB	NA	25 (3– 67)	33 (87%)	NA	17 (45%)	4	NA	NA	NA	NA	NA
Pan et al (n=51)(25)	ChiCTR-IIh-16008711	Lentivirus	CD8	4-1BB	51	23 (2-68)	NA	57	NA	5	NA	4 (8%)	16 (31%)	NA	NA
Park et al (n=53)(26)	NCT01044069	Retrovirus	CD28	CD28	83	44 (23-74)	19 (36%)	63	32 (60%)	NA	6 (11%)	NA	5 (9%)	NA	16 (30%)
Pasquini et al (n=255)(27)	NA	Lentivirus	CD8	4-1BB	255	13 (0.4-26)	71 (28%)	NA	84 (33%)	3	44 (17%)	24 (9%)	NA	46 (18%)	NA
Ravich et al (n=31)(28)	NA	Lentivirus	CD8	4-1BB	33	8 (0.8-24)	4 (13%)	2	15 (48%)	NA	3 (10%)	1 (3%)	3 (10%)	19 (61%)	1 (3%)
Roddie et al (n=20)(29)	NCT02935257	Lentivirus	CD8	4-1BB	25	42 (18-62)	13 (65%)	43	14 (70%)	3	NA	0 (0%)	3 (15%)	NA	6 (30%)
Schultz et al (n=185)(30)	NA	Lentivirus	CD8	4-1BB	200	12 (0-26)	47 (25%)	NA	102 (55%)	NA	NA	13 (7%)	15 (8%)	70 (38%)	10 (5%)
Shah et al (n=55) (31)	NCT02614066 (ZUMA-3)	Retrovirus	CD28	CD28	71	40 (28-52)	24 (44%)	65	50 (91%)	2	NA	0 (0%)	6 (11%)	NA	15 (27%)
Shah et al (n=50)(32)	NCT01593696	NA	CD28	CD28	51	14 (4-30)	22 (44%)	NA	32 (64%)	4	NA	13 (7%)	4 (8%)	NA	NA
Shahid et al (n=35)(33)	NCT01860937	mixed	NA	CD28/4-1BB	48	14 (1-26)	NA	NA	23 (66%)	NA	NA	NA	NA	NA	NA
Wang et al (n=23)(34)	ChiCTR-ONN-16009862; ChiCTR1800019622	Lentivirus	NA	4-1BB	25	42 (10-67)	5 (22%)	48.6	23 (100%)	2	NA	3 (13%)	3 (13%)	10 (43%)	7 (30%)
Wayne et al (n=24)(35)	NCT02625480	Retrovirus	CD28	CD28	31	13 (3-20)	6 (25%)	37	24 (100%)	NA	NA	NA	NA	NA	NA
Yang et al (n=10)(36)	NCT03825718	NA	NA	NA	10	21 (15-39)	NA	9	5 (50%)	NA	NA	NA	NA	9 (90%)	2 (20%)
Zhang et al (n=10)(37)	NA	NA	NA	NA	10	20 (14-39)	NA	NA	4 (40%)	NA	NA	NA	NA	6 (60%)	3 (30%)
Zhang et al (n=254)(38)	NCT03173417; NCT02546739; NCT03671460; ChiCTR1800016541; ChiCTR-ONC-17012829	Lentivirus	NA	CD28/4-1BB	279	NA (1-61)	24 (9%)	NA	158 (62%)	NA	NA	NA	NA	125 (49%)	36 (14%)

Table S6. Additional study characteristics - Intervention

ID	Conditioning regimen	Cyclophosphamide bulk dose (mg/m2)	N dropped due to manufacturing failure	N dropped due to disease progression
An et al (n=47)(1)	Fludarabine combination	NA	4	0
Cao et al (n=15)(2)	Fludarabine /Cyclophosphamide	750	0	1
Chang et al (n=102)(3)	Fludarabine /Cyclophosphamide	NA	NA	NA
Cheng et al (n=6)(4)	Fludarabine/Cyclophosphamide	200-500	0	0
Del Bufalo et al (n=15)(5)	NA	NA	0	0
Frey et al (n=35)(6)	Fludarabine /Cyclophosphamide	1800	1	10
Gardner et al (n=43)(7)	Fludarabine /Cyclophosphamide	2000-4000	4	0
Ghorashian et al (n=14)(8)	Fludarabine /Cyclophosphamide	1500	3	0
Gu et al (n=20)(9)	Fludarabine /Cyclophosphamide	700	1	NA
Han et al (n= 29)(10)	Fludarabine /Cyclophosphamide	1200-1600	0	NA
Hay et al (n=53)(11)	Fludarabine /Cyclophosphamide	NA	0	2
Heng et al (n=10)(12)	Fludarabine /Cyclophosphamide	900	0	0
Hu et al (n=14)(13)	Fludarabine combination	NA	0	2
Jacoby et al (n=20)(14)	Fludarabine /Cyclophosphamide	900	1	0
Jiang et al (n=58)(15)	Fludarabine /Cyclophosphamide	1500	1	1
Kadauke et al (n=70)(16)	Fludarabine /Cyclophosphamide	1000	1	8
Ma et al (n=10)(17)	Fludarabine /Cyclophosphamide	900	0	2
Maschan et al (n=27)(18)	Fludarabine /Cyclophosphamide	750	NA	NA
Maude et al (n=22)(19)	Fludarabine /Cyclophosphamide	NA	NA	NA
Maude et al (n=58)(20)	Fludarabine /Cyclophosphamide	3000-3600	5	6
Maude et al (n=30)(21)	Fludarabine combinations	<1500	NA	NA
Maude et al (n=75)(22)	NA	3000-3600	7	7
Myers et al (n=41)(23)	Fludarabine /Cyclophosphamide	1000	1	1
Ortíz et al (n=38) (24)	Fludarabine /Cyclophosphamide	900	NA	NA
Pan et al (n=51)(25)	Fludarabine /Cyclophosphamide	750	NA	NA
Park et al (n=53)(26)	Fludarabine /Cyclophosphamide	3000	2	7
Pasquini et al (n=255)(27)	Fludarabine /Cyclophosphamide	NA	NA	NA

Ravich et al (n=31)(28)	Fludarabine /Cyclophosphamide	900-1000	NA	2
Roddie et al (n=20)(29)	Fludarabine /Cyclophosphamide	3000-3600	1	4
Schultz et al (n=185)(30)	Fludarabine /Cyclophosphamide	1000	6	5
Shah et al (n=55) (31)	NA	900	1	NA
Shah et al (n=50)(32)	Fludarabine /Cyclophosphamide	2400	0	1
Shahid et al (n=35)(33)	Fludarabine /Cyclophosphamide	1500-3000	1	1
Wang et al (n=23)(34)	Fludarabine	NA	0	0
Wayne et al (n=24)(35)	Fludarabine /Cyclophosphamide	900	4	1
Yang et al (n=10)(36)	Fludarabine /Cyclophosphamide	750	NA	NA
Zhang et al (n=10)(37)	Fludarabine /Cyclophosphamide	NA	NA	NA
Zhang et al (n=254)(38)	Fludarabine /Cyclophosphamide	750	3	14

Table S7. Additional study characteristics - Results

ID	Overall response	N received HSCT	Median follow up	N relapsed	CRS grade 3 or 4	CRS grade 1 or 2	ICANS	ICANS grade 3 or 4	CRS scale
An et al (n=47)(1)	38/47	10	NA	NA	11	28	2	NA	Lee
Cao et al (n=15)(2)	13/14	2	8.02	NA	3	12	1	NA	Lee
Chang et al (n=102)(3)	88/102	NA	7	NA	3	73	NA	NA	NA
Cheng et al (n=6)(4)	4/6	NA	NA	NA	0	1	0	NA	Lee
Del Bufalo et al (n=15)(5)	13/15	4	NA	4	1	9	NA	NA	NA
Frey et al (n=35)(6)	24/35	9	13	NA	25	8	14	2	Penn
Gardner et al (n=43)(7)	40/43	11	9.6	2	18	22	21	9	NA
Ghorashian et al (n=14)(8)	12/14	NA	14.4	6	0	13	7	1	Lee
Gu et al (n=20)(9)	18/22	14	10.09	8	9	10	13	8	Lee
Han et al (n= 29)(10)	26/29	NA	NA	2	6	13	NA	NA	Lee
Hay et al (n=53)(11)	45/53	18	30.9	22	10	30	NA	12	Lee
Heng et al (n=10)(12)	10/10	2	NA	NA	4	6	4	2	MSKCC
Hu et al (n=14)(13)	12/14	4	4.7	6	6	9	5	NA	Lee
Jacoby et al (n=20)(14)	18/20	14	9	4	4	12	11	6	Lee
Jiang et al (n=58)(15)	51/58	21	7.7	22	22	NA	9	9	Lee
Kadauke et al (n=70)(16)	66/70	6	24	10	12	40	19	5	Penn
Ma et al (n=10)(17)	7/10	0	8.7	4	4	6	6	3	Penn
Maschan et al (n=27)(18)	24/27	3	17	16	2	15	13	4	Lee
Maude et al (n=22)(19)	22/22	3	14	4	NA	NA	NA	NA	NA
Maude et al (n=58)(20)	29/58	NA	NA	9	13	20	13	3	Penn
Maude et al (n=30)(21)	27/30	3	7	7	8	22	13	NA	Penn
Maude et al (n=75)(22)	61/75	8	13.1	17	35	23	30	10	Penn
Myers et al (n=41)(23)	40/41	4	34.6	12	6	31	17	NA	Penn
Ortíz et al (n=38) (24)	32/38	NA	5.5	NA	5	NA	2	1	Lee
Pan et al (n=51)(25)	45/49	27	6.8	9	NA	NA	16	NA	Lee
Park et al (n=53)(26)	44/53	17	29	17	14	31	23	22	MSKCC
Pasquini et al (n=255)(27)	213/249	55	13.4	89	41	99	69	23	ASTCT
Ravich et al (n=31)(28)	25/30	4	12.9	12	6	13	9	3	ASTCT
Roddie et al (n=20)(29)	17/20	3	21.7	7	0	11	4	3	ASTCT
Schultz et al (n=185)(30)	156/197	41	11.2	57	39	77	26	12	ASTCT

Shah et al (n=55) (31)	39/55	10	16.4	12	13	36	33	14	Lee
Shah et al (n=50)(32)	31/50	21	57.6	12	9	26	10	4	Lee
Shahid et al (n=35)(33)	27/33	20	46		7	NA	NA	8	ASTCT
Wang et al (n=23)(34)	19/23	5	14	7	5	18	NA	NA	Lee
Wayne et al (n=24)(35)	16/22	10	13.2	NA	7	14	15	6	Lee
Yang et al (n=10)(36)	10/10	5	2.9	0	1	9	0	0	NA
Zhang et al (n=10)(37)	10/10	3	NA	NA	4	5	3	NA	NA
Zhang et al (n=254)(38)	231/254	184	12	43	26	147	NA	17	Lee

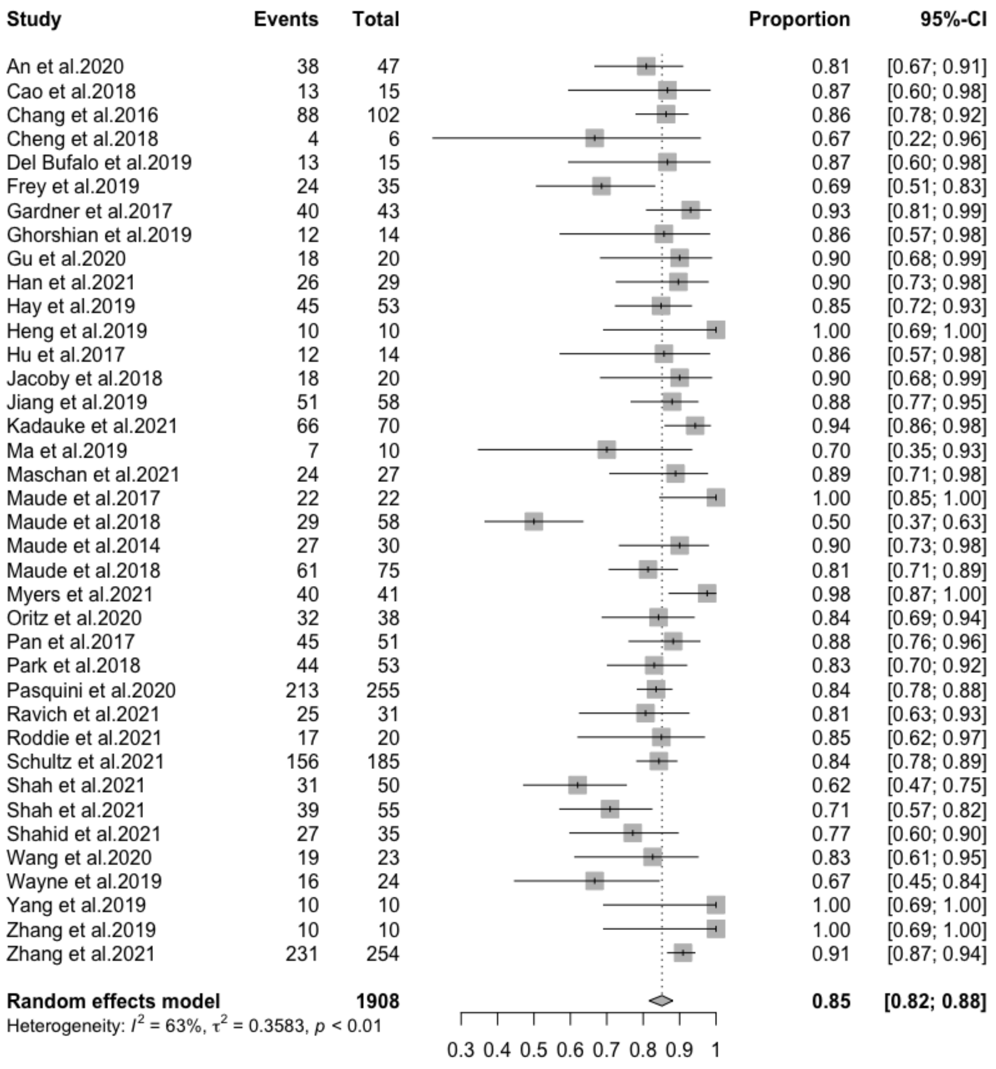

Figure S3. overall response to CAR T in the infused patients

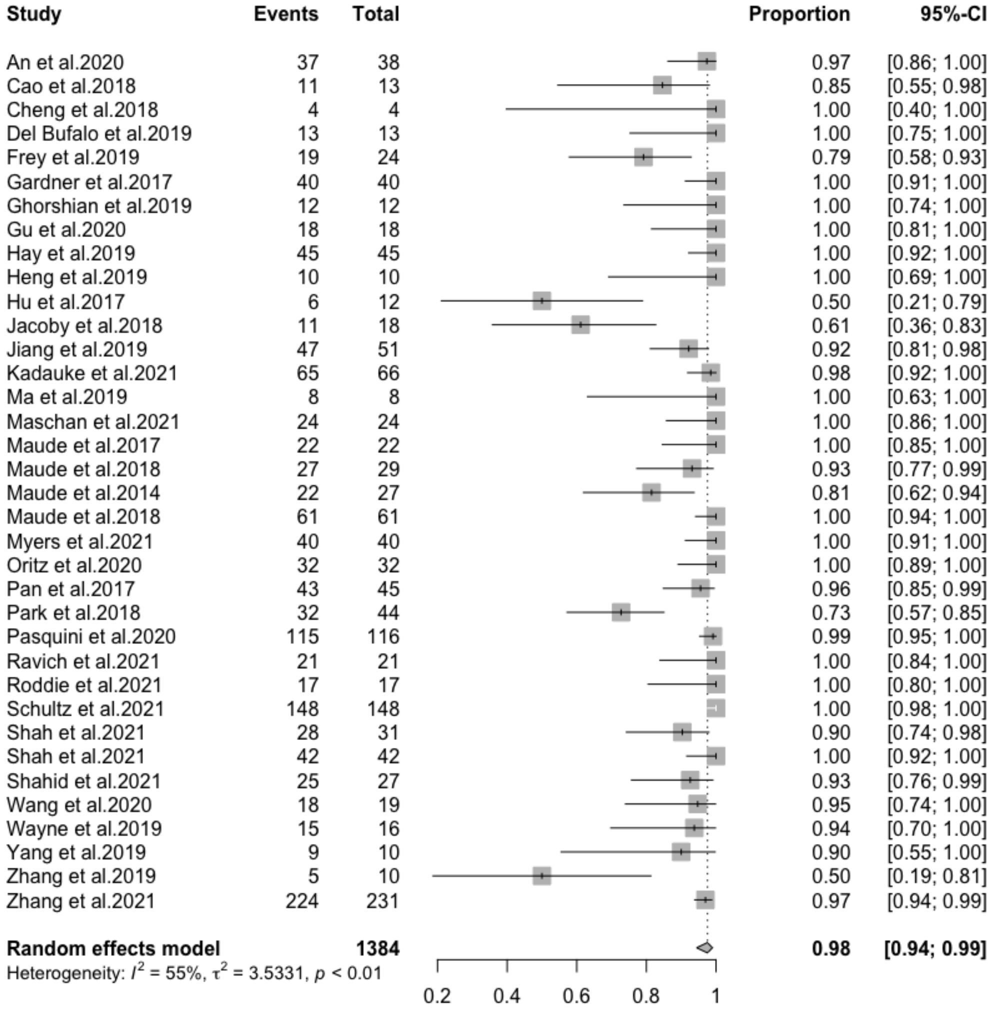

Figure S4. MRD negativity in the infused patients

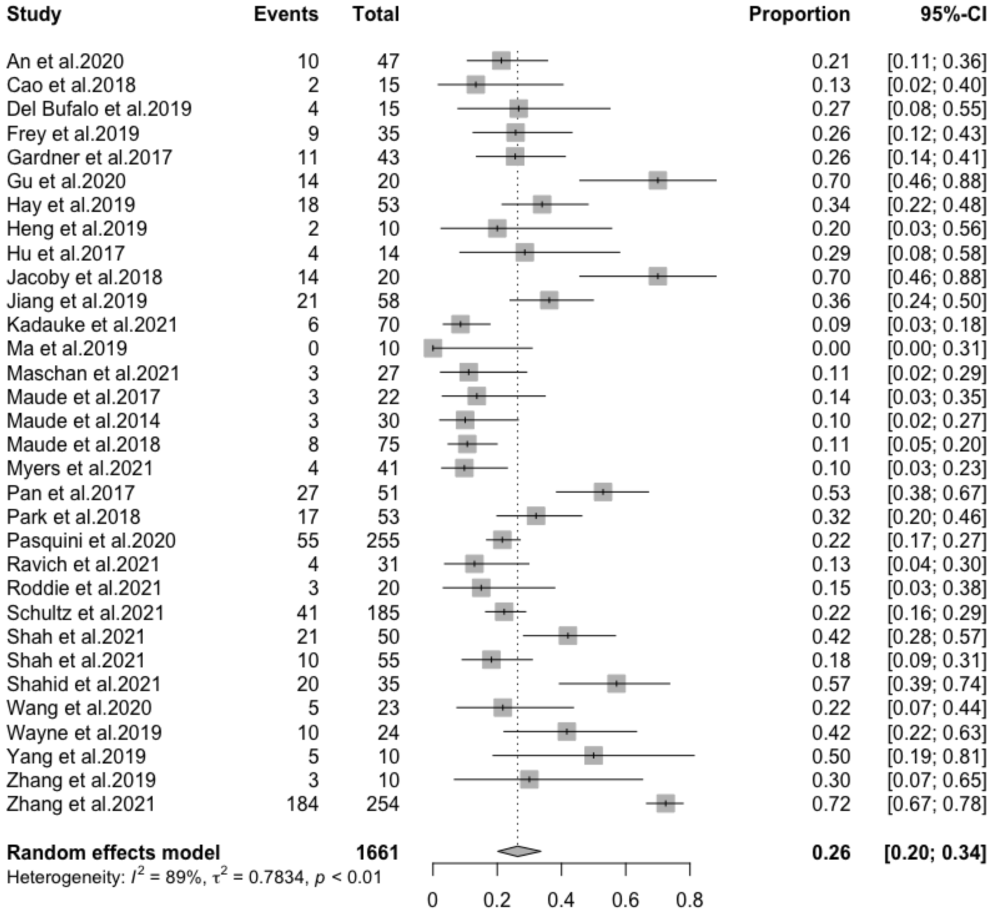

Figure S5. Proportion of infused patients that proceeded to hematopoietic stem cell transplantation after infusion of CAR

Study	Events	Total	Proportion	95%-CI
An et al.2020	39	47	0.83	[0.69; 0.92]
Cao et al.2018	15	15	1.00	[0.78; 1.00]
Chang et al.2016	76	102	0.75	[0.65; 0.83]
Cheng et al.2018	1	6	0.17	[0.00; 0.64]
Del Bufalo et al.2019	10	15	0.67	[0.38; 0.88]
Frey et al.2019	33	35	0.94	[0.81; 0.99]
Gardner et al.2017	40	43	0.93	[0.81; 0.99]
Ghorshian et al.2019	13	14	0.93	[0.66; 1.00]
Gu et al.2020	19	20	0.95	[0.75; 1.00]
Han et al.2021	19	29	0.66	[0.46; 0.82]
Hay et al.2019	40	53	0.75	[0.62; 0.86]
Heng et al.2019	10	10	1.00	[0.69; 1.00]
Hu et al.2017	15	15	1.00	[0.78; 1.00]
Jacoby et al.2018	16	20	0.80	[0.56; 0.94]
Kadauke et al.2021	52	70	0.74	[0.62; 0.84]
Ma et al.2019	10	10	1.00	[0.69; 1.00]
Maschan et al.2021	17	27	0.63	[0.42; 0.81]
Maude et al.2018	33	58	0.57	[0.43; 0.70]
Maude et al.2014	30	30	1.00	[0.88; 1.00]
Maude et al.2018	58	75	0.77	[0.66; 0.86]
Myers et al.2021	37	41	0.90	[0.77; 0.97]
Park et al.2018	45	53	0.85	[0.72; 0.93]
Pasquini et al.2020	140	255	0.55	[0.49; 0.61]
Ravich et al.2021	19	31	0.61	[0.42; 0.78]
Roddie et al.2021	11	20	0.55	[0.32; 0.77]
Schultz et al.2021	116	185	0.63	[0.55; 0.70]
Shah et al.2021	35	50	0.70	[0.55; 0.82]
Shah et al.2021	49	55	0.89	[0.78; 0.96]
Wang et al.2020	23	23	1.00	[0.85; 1.00]
Wayne et al.2019	21	24	0.88	[0.68; 0.97]
Yang et al.2019	10	10	1.00	[0.69; 1.00]
Zhang et al.2019	9	10	0.90	[0.55; 1.00]
Zhang et al.2021	173	254	0.68	[0.62; 0.74]
**Random effects model**		**1705**	**0.83**	**[0.76; 0.89]**

Heterogeneity: $I^2 = 68\%$, $\tau^2 = 1.1478$, $p < 0.01$

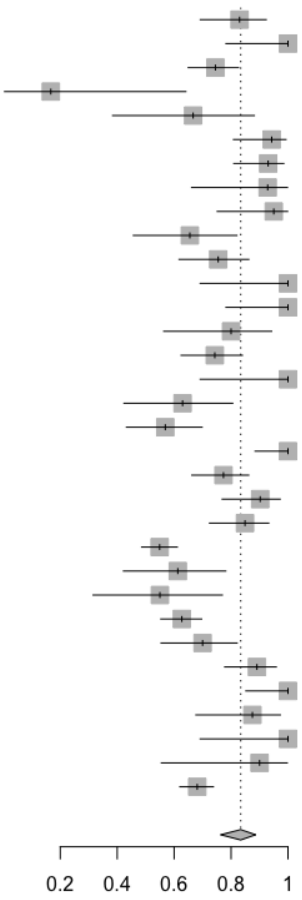

Figure S6. Infused patients who suffered cytokine release syndrome of any grade after CAR T infusion

Study	Events	Total	Proportion	95%-CI
An et al.2020	11	47	0.23	[0.12; 0.38]
Cao et al.2018	3	15	0.20	[0.04; 0.48]
Chang et al.2016	3	102	0.03	[0.01; 0.08]
Cheng et al.2018	0	6	0.00	[0.00; 0.46]
Del Bufalo et al.2019	1	15	0.07	[0.00; 0.32]
Frey et al.2019	25	35	0.71	[0.54; 0.85]
Gardner et al.2017	18	43	0.42	[0.27; 0.58]
Ghorshian et al.2019	0	14	0.00	[0.00; 0.23]
Gu et al.2020	9	20	0.45	[0.23; 0.68]
Han et al.2021	6	29	0.21	[0.08; 0.40]
Hay et al.2019	10	53	0.19	[0.09; 0.32]
Heng et al.2019	4	10	0.40	[0.12; 0.74]
Hu et al.2017	6	15	0.40	[0.16; 0.68]
Jacoby et al.2018	4	20	0.20	[0.06; 0.44]
Jiang et al.2019	22	58	0.38	[0.26; 0.52]
Kadauke et al.2021	12	70	0.17	[0.09; 0.28]
Ma et al.2019	4	10	0.40	[0.12; 0.74]
Maschan et al.2021	2	27	0.07	[0.01; 0.24]
Maude et al.2018	13	58	0.22	[0.13; 0.35]
Maude et al.2014	8	30	0.27	[0.12; 0.46]
Maude et al.2018	35	75	0.47	[0.35; 0.59]
Myers et al.2021	6	41	0.15	[0.06; 0.29]
Oritz et al.2020	5	38	0.13	[0.04; 0.28]
Park et al.2018	14	53	0.26	[0.15; 0.40]
Pasquini et al.2020	41	255	0.16	[0.12; 0.21]
Ravich et al.2021	6	31	0.19	[0.07; 0.37]
Roddie et al.2021	0	20	0.00	[0.00; 0.17]
Schultz et al.2021	39	185	0.21	[0.15; 0.28]
Shah et al.2021	9	50	0.18	[0.09; 0.31]
Shah et al.2021	13	55	0.24	[0.13; 0.37]
Shahid et al.2021	7	35	0.20	[0.08; 0.37]
Wang et al.2020	5	23	0.22	[0.07; 0.44]
Wayne et al.2019	7	24	0.29	[0.13; 0.51]
Yang et al.2019	1	10	0.10	[0.00; 0.45]
Zhang et al.2019	4	10	0.40	[0.12; 0.74]
Zhang et al.2021	26	254	0.10	[0.07; 0.15]
**Random effects model**		**1836**	**0.21**	**[0.16; 0.26]**

Heterogeneity: $I^2 = 75\%$, $\tau^2 = 0.5730$, $p < 0.01$

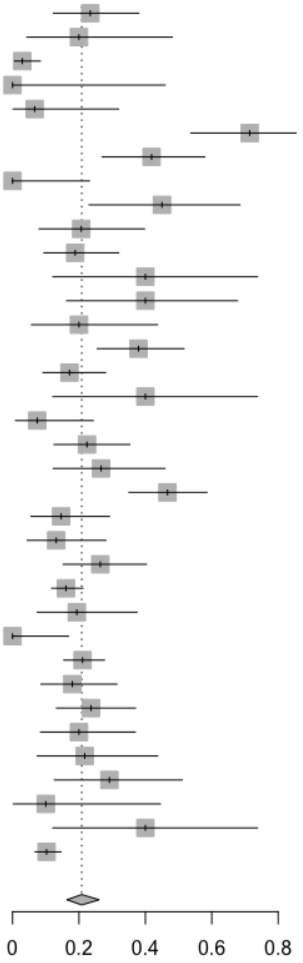

Figure S7. Incidence of cytokine release syndrome of grade 3 or higher in infused patients

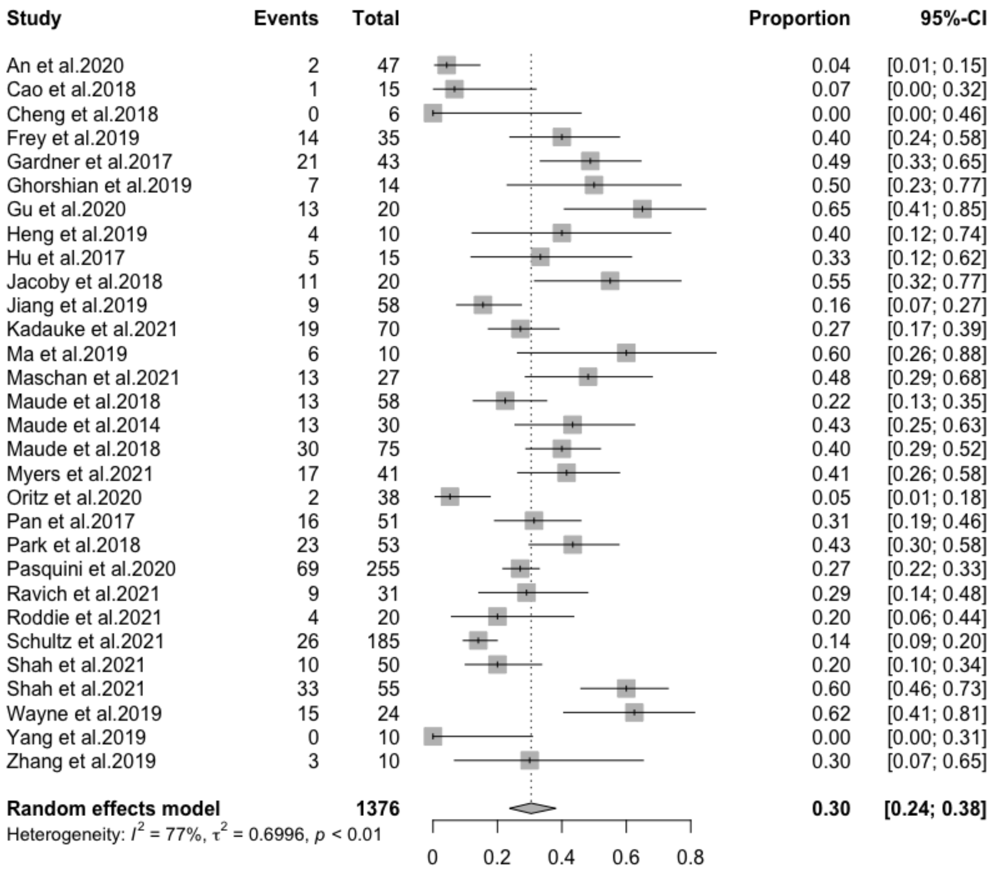

Figure S8. Neurotoxicity in the infused patients

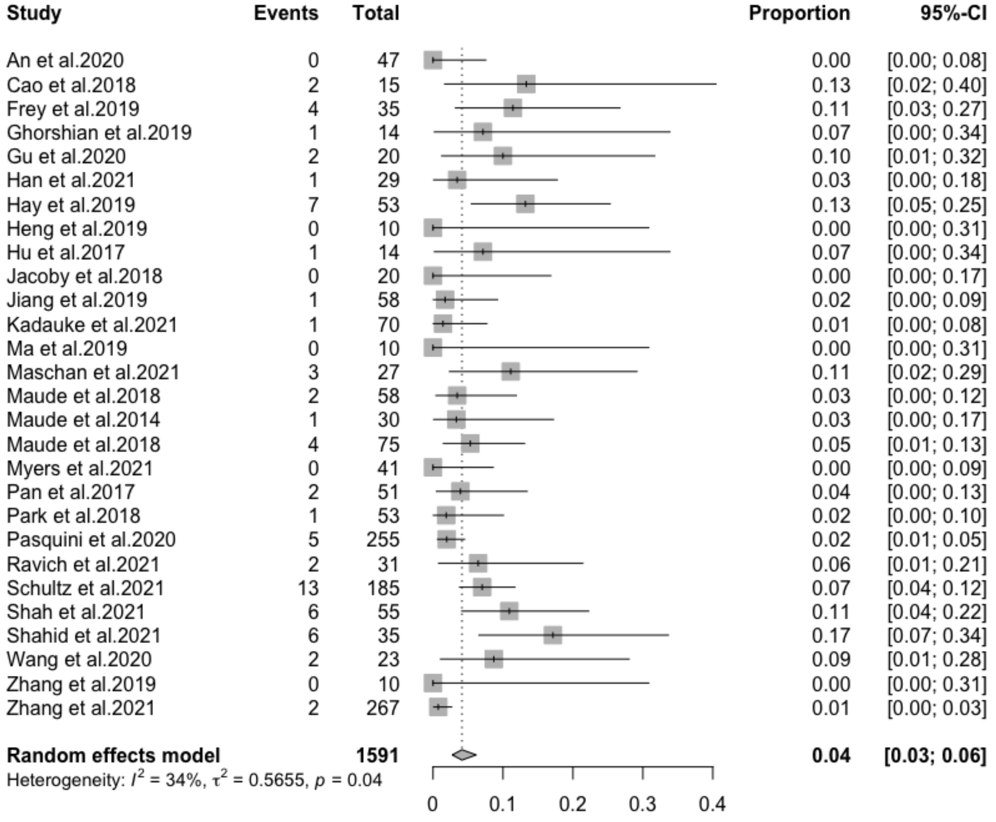

Figure S9. treatment related mortality in R/R ALL patients infused with CD19 CAR T cells

Table S8. Univariate cox regression analysis of OS and EFS

Variable	Group	HR for overall survival			HR for event free survival		
		HR	95% CI	p value	HR	95% CI	p value
Age	Mixed	0.95	0.73 - 1.24	0.707	1.11	0.85 - 1.46	0.43
Domain	41BB	0.56	0.46 - 0.70	<0.001	0.57	0.46 - 0.70	<0.001
Disease morphology	Remission	0.51	0.41 - 0.64	<0.001	0.73	0.61 - 0.88	<0.001
Cyclophosphamide	Low	0.56	0.46 - 0.67	<0.001	0.66	0.55 - 0.79	<0.001
Study type	Trial	1.32	1.06 - 1.63	0.0127	0.87	0.74 - 1.04	0.118
Start date		0.90	0.85 - 0.94	<0.001	0.93	0.90 - 0.97	<0.001

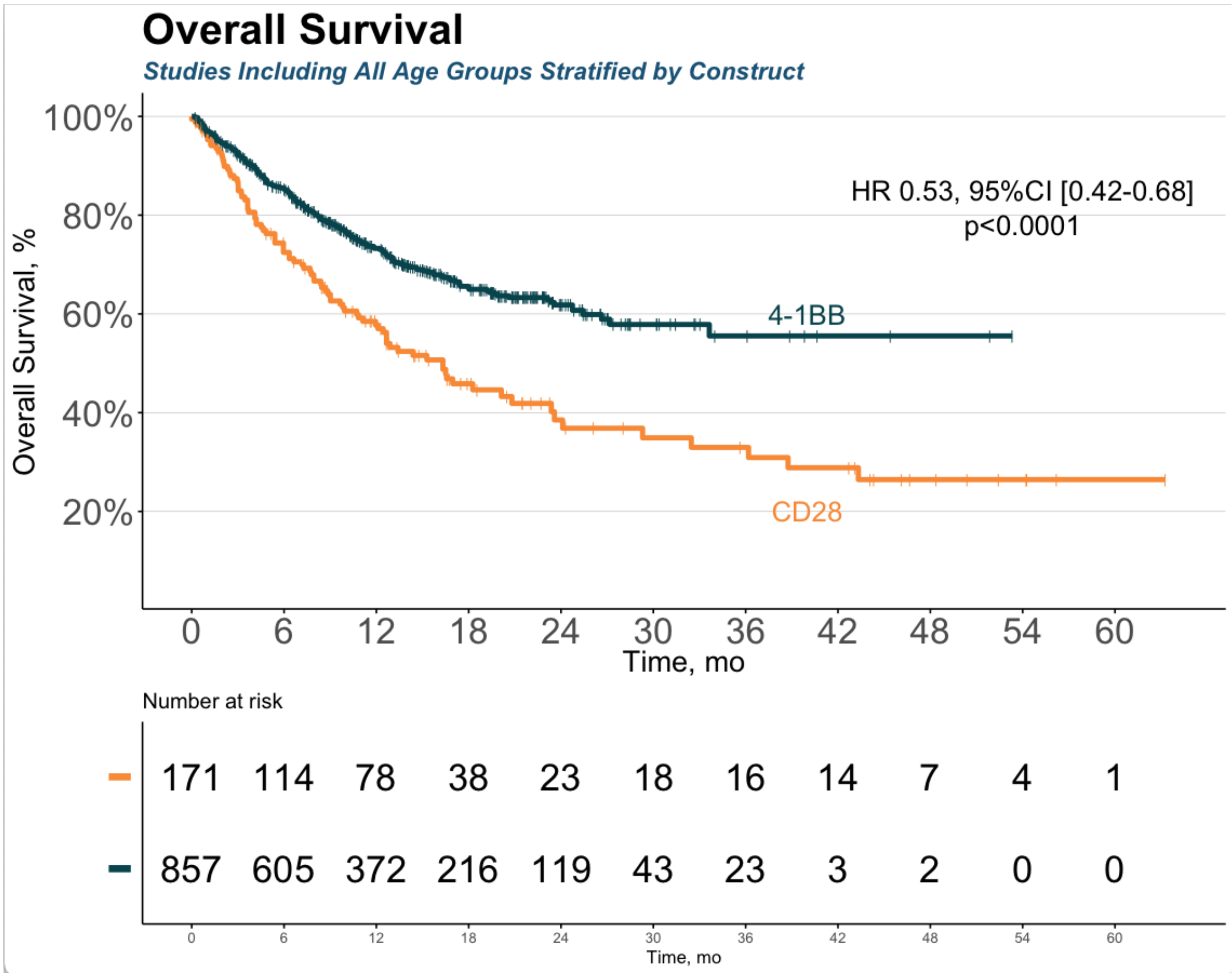

Figure S10 Difference in Overall survival between CD28 and 4-1BB signaling domains in reports that included patients of any age (pediatric and adult)

References:

1. An F, Wang H, Liu Z, Wu F, Zhang J, Tao Q, et al. Influence of patient characteristics on chimeric antigen receptor T cell therapy in B-cell acute lymphoblastic leukemia. Nat Commun. 2020 Dec;11(1):5928.

2. Cao J, Wang G, Cheng H, Wei C, Qi K, Sang W, et al. Potent anti-leukemia activities of humanized CD19-targeted Chimeric antigen receptor T (CAR-T) cells in patients with relapsed/refractory acute lymphoblastic leukemia. Am J Hematol. 2018 Jul;93(7):851–8.

3. Chang LJ, Dong L, Liu YC, Tsao ST, Li YC, Liu L, et al. Safety and Efficacy Evaluation of 4SCAR19 Chimeric Antigen Receptor-Modified T Cells Targeting B Cell Acute Lymphoblastic Leukemia - Three-Year Follow-up of a Multicenter Phase I/II Study. Blood. 2016 Dec 2;128(22):587–587.

4. Cheng Z, Wei R, Ma Q, Shi L, He F, Shi Z, et al. In Vivo Expansion and Antitumor Activity of Coinfused CD28- and 4-1BB-Engineered CAR-T Cells in Patients with B Cell Leukemia. Mol Ther. 2018 Apr;26(4):976–85.

5. Del Bufalo F, Quintarelli C, De Angelis B, Caruana I, Sinibaldi M, Vinti L, et al. Academic, Phase I/II Trial on T Cells Expressing a Second Generation, CD19-Specific Chimeric Antigen Receptor (CAR) and Inducible Caspase 9 Safety Switch for the Treatment of B-Cell Precursor Acute Lymphoblastic Leukemia (BCP-ALL) and B-Cell Non-Hodgkin Lymphoma (B-NHL) in Children. Blood. 2019 Nov 13;134(Supplement_1):1341–1341.

6. Frey NV, Shaw PA, Hexner EO, Pequignot E, Gill S, Luger SM, et al. Optimizing Chimeric Antigen Receptor T-Cell Therapy for Adults With Acute Lymphoblastic Leukemia. J Clin Oncol. 2020 Feb 10;38(5):415–22.

7. Gardner RA, Finney O, Annesley C, Brakke H, Summers C, Leger K, et al. Intent-to-treat leukemia remission by CD19 CAR T cells of defined formulation and dose in children and young adults. Blood. 2017 Jun;129(25):3322–31.

8. Ghorashian S, Kramer AM, Onuoha S, Wright G, Bartram J, Richardson R, et al. Enhanced CAR T cell expansion and prolonged persistence in pediatric patients with ALL treated with a low-affinity CD19 CAR. Nat Med. 2019 Sep;25(9):1408–14.

9. Gu R, Liu F, Zou D, Xu Y, Lu Y, Liu B, et al. Efficacy and safety of CD19 CAR T constructed with a new anti-CD19 chimeric antigen receptor in relapsed or refractory acute lymphoblastic leukemia. J Hematol OncolJ Hematol Oncol. 2020 Dec;13(1):122.

10. Han L, Zhao L, You H, Gao Q, Zhou J, Zhou K, et al. Culturing adequate CAR-T cells from less peripheral blood to treat B-cell malignancies. Cancer Biol Med. 2021;18(4):1066–79.

11. Hay KA, Gauthier J, Hirayama AV, Voutsinas JM, Wu Q, Li D, et al. Factors associated with durable EFS in adult B-cell ALL patients achieving MRD-negative CR after CD19 CAR T-cell therapy. Blood. 2019 Apr 11;133(15):1652–63.

12. Heng G, Jia J, Li S, Fu G, Wang M, Qin D, et al. Sustained Therapeutic Efficacy of Humanized Anti-CD19 Chimeric Antigen Receptor T Cells in Relapsed/Refractory Acute Lymphoblastic Leukemia. Clin Cancer Res. 2020 Apr 1;26(7):1606–15.

13. Hu Y, Wu Z, Luo Y, Shi J, Yu J, Pu C, et al. Potent Anti-leukemia Activities of Chimeric Antigen Receptor–Modified T Cells against CD19 in Chinese Patients with Relapsed/Refractory Acute Lymphocytic Leukemia. Clin Cancer Res. 2017 Jul 1;23(13):3297–306.

14. Jacoby E, Bielorai B, Avigdor A, Itzhaki O, Hutt D, Nussboim V, et al. Locally produced CD19 CAR T cells leading to clinical remissions in medullary and extramedullary relapsed acute lymphoblastic leukemia. Am J Hematol. 2018 Dec;93(12):1485–92.

15. Jiang H, Li C, Yin P, Guo T, Liu L, Xia L, et al. Anti-CD19 chimeric antigen receptor-modified T-cell therapy bridging to allogeneic hematopoietic stem cell transplantation for relapsed/refractory B-cell acute lymphoblastic leukemia: an open-label pragmatic clinical trial. Am J Hematol. 2019;94(10):1113–22.

16. Kadauke S, Myers RM, Li Y, Aplenc R, Baniewicz D, Barrett DM, et al. Risk-Adapted Preemptive Tocilizumab to Prevent Severe Cytokine Release Syndrome After CTL019 for Pediatric B-Cell Acute Lymphoblastic Leukemia: A Prospective Clinical Trial. J Clin Oncol. 2021 Jan 8;Jco2002477.

17. Ma F, Ho J, Du H, Xuan F, Wu X, Wang Q, et al. Evidence of long-lasting anti-CD19 activity of engrafted CD19 chimeric antigen receptor–modified T cells in a phase I study targeting pediatrics with acute lymphoblastic leukemia. Hematol Oncol. 2019 Dec;37(5):601–8.

18. Maschan M, Molostova O, Shelikhova L, Pershin D, Muzalevskii Y, Reese-Koc J, et al. Multiple site place-of-care manufactured anti-CD19 CAR-T cells induce high remission rates in B-cell malignancy patients. Nat Commun. 2021;12(1):7200.

19. Maude SL, Hucks GE, Callahan C, Baniewicz D, Fasano C, Barker C, et al. Durable Remissions with Humanized CD19-Targeted Chimeric Antigen Receptor (CAR)-Modified T Cells in CAR-Naive and CAR-Exposed Children and Young Adults with Relapsed/Refractory Acute Lymphoblastic Leukemia. Blood. 2017 Dec;130.

20. Maude SL, Grupp SA, Mody R, Driscoll T, Laetsch TW, Qayed M, et al. An updated analysis of tisagenlecleucel in pediatric/ young adult patients with relapsed/refractory (R/R) B-cell acute lymphoblastic leukemia (B-ALL) in a us multicenter clinical trial (ENSIGN). Hemasphere. 2018 Jun;2 (Supplement 2):41.

21. Maude SL, Frey N, Shaw PA, Aplenc R, Barrett DM, Bunin NJ, et al. Chimeric antigen receptor T cells for sustained remissions in leukemia. N Engl J Med. 2014 Oct 16;371(16):1507–17.

22. Maude S, Laetsch T, Buechner J, Rives S, Boyer M, Bittencourt H, et al. Tisagenlecleucel in Children and Young Adults with B-Cell Lymphoblastic Leukemia. N Engl J Med. 2018;378(5):439–48.

23. Myers RM, Barz Leahy A, Callahan C, Fasano CC, Baniewicz D, Li Y, et al. Humanized CD19-Targeted Chimeric Antigen Receptor (CAR) T Cells in CAR-Naive and CAR-Exposed Children and Young Adults With Relapsed or Refractory Acute Lymphoblastic Leukemia. J Clin Oncol Off J Am Soc Clin Oncol. 2021;39(27):3044–55.

24. Ortíz-Maldonado V, Rives S, Castellà M, Alonso-Saladrigues A, Benítez-Ribas D, Caballero-Baños M, et al. CART19-BE-01: A Multicenter Trial of ARI-0001 Cell Therapy in Patients with CD19+ Relapsed/Refractory Malignancies. Mol Ther. 2021 Feb;29(2):636–44.

25. Pan J, Yang JF, Deng BP, Zhao XJ, Zhang X, Lin YH, et al. High efficacy and safety of low-dose CD19-directed CAR-T cell therapy in 51 refractory or relapsed B acute lymphoblastic leukemia patients. Leukemia. 2017 Dec;31(12):2587–93.

26. Park JH, Rivière I, Gonen M, Wang X, Sénéchal B, Curran KJ, et al. Long-Term Follow-up of CD19 CAR Therapy in Acute Lymphoblastic Leukemia. N Engl J Med. 2018 Feb 1;378(5):449–59.

27. Pasquini MC, Hu ZH, Curran K, Laetsch T, Locke F, Rouce R, et al. Real-world evidence of tisagenlecleucel for pediatric acute lymphoblastic leukemia and non-Hodgkin lymphoma. Blood Adv. 2020 Nov 10;4(21):5414–24.

28. Ravich JW, Huang S, Zhou Y, Brown P, Pui CH, Inaba H, et al. Impact of High Disease Burden on Survival in Pediatric Patients with B-ALL Treated with Tisagenlecleucel. Transplant Cell Ther. 2021 Dec 4;

29. Roddie C, Dias J, O'Reilly MA, Abbasian M, Cadinanos-Garai A, Vispute K, et al. Durable Responses and Low Toxicity After Fast Off-Rate CD19 Chimeric Antigen Receptor-T Therapy in Adults With Relapsed or Refractory B-Cell Acute Lymphoblastic Leukemia. J Clin Oncol Off J Am Soc Clin Oncol. 2021 Oct 20;39(30):3352–63.

30. Schultz LM, Baggott C, Prabhu S, Pacenta HL, Phillips CL, Rossoff J, et al. Disease Burden Affects Outcomes in Pediatric and Young Adult B-Cell Lymphoblastic Leukemia After Commercial Tisagenlecleucel: A Pediatric Real-World Chimeric Antigen Receptor Consortium Report. J Clin Oncol Off J Am Soc Clin Oncol. 2021 Dec 9;JCO2003585.

31. Shah BD, Ghobadi A, Oluwole OO, Logan AC, Boissel N, Cassaday RD, et al. KTE-X19 for relapsed or refractory adult B-cell acute lymphoblastic leukaemia: phase 2 results of the single-arm, open-label, multicentre ZUMA-3 study. The Lancet. 2021 Aug;398(10299):491–502.

32. Shah NN, Lee DW, Yates B, Yuan CM, Shalabi H, Martin S, et al. Long-Term Follow-Up of CD19-CAR T-Cell Therapy in Children and Young Adults With B-ALL. J Clin Oncol. 2021 Mar 25;JCO.20.02262.

33. Shahid S, Ramaswamy K, Flynn J, Mauguen A, Perica K, Park JH, et al. Impact of Bridging Chemotherapy on Clinical Outcomes of CD19-Specific CAR T Cell Therapy in Children/Young Adults with Relapsed/Refractory B Cell Acute Lymphoblastic Leukemia. Transplant Cell Ther. 2021 Nov 28;

34. Wang J, Mou N, Yang Z, Li Q, Jiang Y, Meng J, et al. Efficacy and safety of humanized anti-CD19-CAR-T therapy following intensive lymphodepleting chemotherapy for refractory/relapsed B acute lymphoblastic leukaemia. Br J Haematol. 2020 Oct;191(2):212–22.

35. Wayne A, Huynh V, Hijiya N, Rouce R, Brown P, Krueger J, et al. PHASE 1 RESULTS OF ZUMA-4: KTE-X19, AN ANTI-CD19 CHIMERIC ANTIGEN RECEPTOR T CELL THERAPY, IN PEDIATRIC AND ADOLESCENT PATIENTS WITH RELAPSED/REFRACTORY B CELL ACUTE LYMPHOBLASTIC LEUKEMIA: PS962. Hemasphere. 2019;3:433.

36. Yang JF, He JP, Zhang X, Wang ZG, Zhang YL, Cai SB, et al. A Feasibility and Safety Study of a New CD19-Directed Fast CAR-T Therapy for Refractory and Relapsed B Cell Acute Lymphoblastic Leukemia. Blood. 2019 Nov;134.

37. Zhang X, Lu XA, Yang J, Zhang G, Li J, Song L, et al. Efficacy and safety of anti-CD19 CAR T-cell therapy in 110 patients with B-cell acute lymphoblastic leukemia with high-risk features. Blood Adv. 2020 May 26;4(10):2325–38.

38. Zhang X, Yang J, Li J, Li W, Song D, Lu XA, et al. Factors associated with treatment response to CD19 CAR-T therapy among a large cohort of B cell acute lymphoblastic leukemia. Cancer Immunol Immunother CII. 2021 Aug 7;
